# [μ-Bis(diphenyl­phosphan­yl)ethane-1:2κ^2^ *P*:*P*′]nona­carbonyl-1κ^3^ *C*,2κ^3^ *C*,3κ^3^ *C*-(triphenyl­stibine-3κ*Sb*)-*triangulo*-triruthenium(0)

**DOI:** 10.1107/S1600536810054218

**Published:** 2011-01-15

**Authors:** Omar bin Shawkataly, Imthyaz Ahmed Khan, Siti Syaida Sirat, Chin Sing Yeap, Hoong-Kun Fun

**Affiliations:** a Chemical Sciences Programme, School of Distance Education, Universiti Sains Malaysia, 11800 USM, Penang, Malaysia; b X-ray Crystallography Unit, School of Physics, Universiti Sains Malaysia, 11800 USM, Penang, Malaysia

## Abstract

The asymmetric unit of the title *triangulo*-triruthenium compound, [Ru_3_(C_26_H_24_P_2_)(C_18_H_15_Sb)(CO)_9_], consists of two crystallographically independent mol­ecules, *A* and *B*. The bis­(diphenyl­phosphan­yl)ethane ligand bridges an Ru—Ru bond and the monodentate stibine ligand bonds to the third Ru atom. Both the stibine and phosphine ligands are equatorial with respect to the Ru_3_ triangle. Additionally, each Ru atom carries one equatorial and two axial terminal carbonyl ligands. The three stibine-substituted benzene rings make dihedral angles of 38.7 (3), 71.5 (3) and 70.0 (3)° with each other in mol­ecule *A* whereas these angles are 83.9 (3), 88.2 (3) and 56.8 (3)° in mol­ecule *B*. Similarly, the dihedral angles between the two benzene rings are 80.7 (3) and 87.6 (3)° for the two diphenyl­phosphanyl groups in mol­ecule *A* and 84.0 (3) and 72.6 (4)° in mol­ecule *B*. In the crystal, mol­ecules are linked into tetra­mers *via* inter­molecular C—H⋯O hydrogen bonds. Weak inter­molecular C—H⋯π inter­actions further stabilize the crystal structure.

## Related literature

For general background to *triangulo*-triruthenium derivatives, see: Bruce *et al.* (1985 ▶, 1988*a* ▶,*b* ▶). For related structures, see: Shawkataly *et al.* (1998 ▶, 2004 ▶, 2010 ▶, 2011 ▶). For the synthesis of Ru_3_(CO)_10_(μ-Ph_2_PCH_2_CH_2_PPh_2_), see: Bruce *et al.* (1983 ▶). For the stability of the temperature controller used in the data collection, see: Cosier & Glazer (1986 ▶).
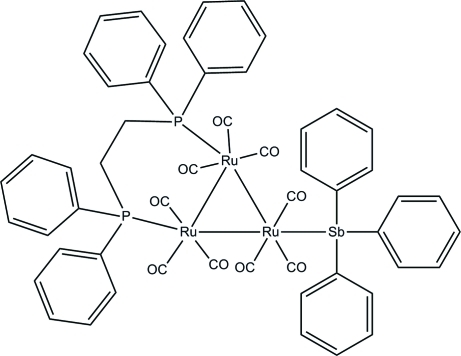


## Experimental

### 

#### Crystal data

[Ru_3_(C_26_H_24_P_2_)(C_18_H_15_Sb)(CO)_9_]
*M* *_r_* = 1306.74
Triclinic, 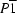

*a* = 15.1589 (8) Å
*b* = 18.6156 (10) Å
*c* = 20.0398 (11) Å
α = 113.233 (1)°
β = 97.986 (1)°
γ = 97.339 (1)°
*V* = 5041.5 (5) Å^3^
*Z* = 4
Mo *K*α radiation
μ = 1.53 mm^−1^
*T* = 100 K
0.51 × 0.15 × 0.08 mm

#### Data collection

Bruker APEXII DUO CCD area-detector diffractometer
Absorption correction: multi-scan (*SADABS*; Bruker, 2009 ▶) *T*_min_ = 0.506, *T*_max_ = 0.885
82542 measured reflections
28970 independent reflections
21778 reflections with *I* > 2σ(*I*)
*R*_int_ = 0.033

#### Refinement

*R*[*F* ^2^ > 2σ(*F* ^2^)] = 0.045
*wR*(*F* ^2^) = 0.121
*S* = 1.11
28970 reflections
1225 parameters
H-atom parameters constrained
Δρ_max_ = 3.47 e Å^−3^
Δρ_min_ = −2.34 e Å^−3^

### 

Data collection: *APEX2* (Bruker, 2009 ▶); cell refinement: *SAINT* (Bruker, 2009 ▶); data reduction: *SAINT*; program(s) used to solve structure: *SHELXTL* (Sheldrick, 2008 ▶); program(s) used to refine structure: *SHELXTL*; molecular graphics: *SHELXTL*; software used to prepare material for publication: *SHELXTL* and *PLATON* (Spek, 2009 ▶).

## Supplementary Material

Crystal structure: contains datablocks global, I. DOI: 10.1107/S1600536810054218/sj5087sup1.cif

Structure factors: contains datablocks I. DOI: 10.1107/S1600536810054218/sj5087Isup2.hkl

Additional supplementary materials:  crystallographic information; 3D view; checkCIF report

## Figures and Tables

**Table 1:** Hydrogen-bond geometry (Å, °) *Cg*1, *Cg*2 and *Cg*3 are the centroids of the C21*B*–C26*B*, C39*B*–C44*B* and C15*A*–C20*A* benzene rings, respectively.

*D*—H⋯*A*	*D*—H	H⋯*A*	*D*⋯*A*	*D*—H⋯*A*
C18*A*—H18*A*⋯O7*B*^i^	0.93	2.55	3.337 (7)	142
C34*A*—H34*A*⋯O2*A*	0.93	2.56	3.375 (7)	147
C34*B*—H34*B*⋯O8*B*^i^	0.93	2.57	3.414 (7)	151
C5*B*—H5*BA*⋯*Cg*1^ii^	0.93	3.00	3.723 (9)	136
C25*B*—H25*B*⋯*Cg*2^iii^	0.93	2.95	3.856 (8)	166
C43*A*—H43*A*⋯*Cg*3^iii^	0.93	2.91	3.624 (6)	135

Symmetry codes: (i) 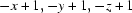; (ii) 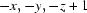; (iii) 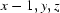.

**Fig. 1.**
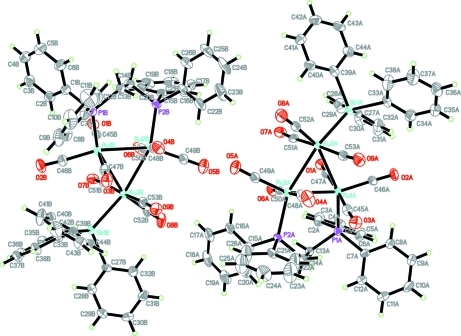
The molecular structure of the title compound with 50% probability ellipsoids for non-H atoms.

**Fig. 2.**
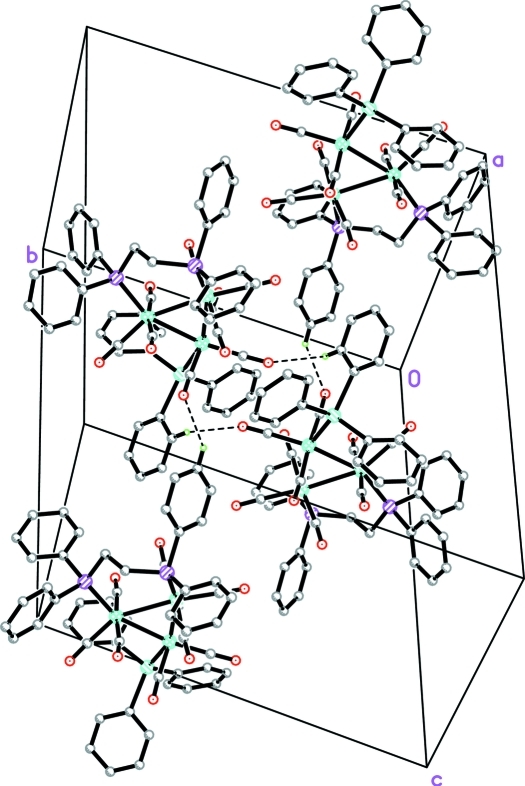
The crystal packing of the title compound, showing the molecules linked into a tetramer. Hydrogen atoms that are not involved in the hydrogen-bonding (dashed lines) have been omitted for clarity.

## supplementary crystallographic information

### Comment

A large number of substituted derivatives, Ru_3_(CO)_12-n_*L*_n_ (*L* = group 15 ligand) have been reported (Bruce *et al.*, 1985, 1988*a*,*b*). As part of our study on the substitution of transition metal-carbonyl clusters with mixed-ligand complexes, we have published several structures of *triangulo*-triruthenium-carbonyl clusters containing mixed P/As and P/Sb ligands (Shawkataly *et al.*, 1998, 2004, 2010, 2011). Herein we report the synthesis and structure of the title compound.

The asymmetric unit of title compound consists of two crystallographically independent molecules, *A* and *B* (Fig. 1). The bis(diphenylphosphanyl)ethane ligand bridges the Ru1–Ru2 bond and the monodentate stibine ligand bonds to the Ru3 atom. Both the stibine and phosphine ligands are equatorial with respect to the Ru_3_ triangle. Additionally, each Ru atom carries one equatorial and two axial terminal carbonyl ligands. The three stibine-substituted benzene rings make dihedral angles (C27–C32/C33–C38, C27–C32/C39–C44 and C33–C38/C39–C44) of 38.7 (3), 71.5 (3) and 70.0 (3) (19)° with each other respectively for molecule *A* whereas these angles are 83.9 (3), 88.2 (3) and 56.8 (3)° for molecule *B*. Similarly, the dihedral angles between the two benzene rings (C1–C6/C7–C12 and C15–C20/C21–C26) are 80.7 (3) and 87.6 (3)° in molecule *A* and 84.0 (3) and 72.6 (4)° in molecule *B* for the two diphenylphosphanyl groups respectively.

In the crystal packing, the molecules are linked together *via* intermolecular C18—H18A···O7B, C34A—H34A···O2A and C34B—H34B···O8B hydrogen bonds into tetramers (Fig. 2, Table 1). Weak intermolecular C—H···π interactions (Table 1) further stabilize the crystal structure.

### Experimental

All manipulations were performed under a dry, oxygen-free nitrogen atmosphere using standard Schlenk techniques. All solvents were dried over sodium and distilled from sodium benzophenone ketyl under dry oxygen free nitrogen. Triphenylstibine (Fluka) is used as received and Ru_3_(CO)_10_(µ-Ph_2_PCH_2_CH_2_PPh_2_) (Bruce *et al.*,1983) was prepared by a reported procedure. The title compound was obtained by refluxing equimolar quantities of Ru_3_(CO)_10_(µ-Ph_2_PCH_2_CH_2_PPh_2_) and triphenylstibine in hexane under a nitrogen atmosphere. Crystals suitable for X-ray diffraction were grown by slow solvent / solvent diffusion of CH_3_OH into CH_2_Cl_3_.

### Refinement

All hydrogen atoms were positioned geometrically and refined using a riding model with C—H = 0.93 or 0.97 Å and *U*_iso_(H) = 1.2*U*_eq_(C). The maximum and minimum residual electron density peaks of 2.13 and -1.16 e Å^-3^ were located 0.85 Å and 1.41 Å, respectively from the Ru3 atom.

### Crystal data

**Table:**

[Ru_3_(C_26_H_24_P_2_)(C_18_H_15_Sb)(CO)_9_]	*Z* = 4
*M**_r_* = 1306.74	*F*(000) = 2568
Triclinic, *P*1	*D*_x_ = 1.722 Mg m^−^^3^
Hall symbol: -P 1	Mo *K*α radiation, λ = 0.71073 Å
*a* = 15.1589 (8) Å	Cell parameters from 9952 reflections
*b* = 18.6156 (10) Å	θ = 2.6–32.6°
*c* = 20.0398 (11) Å	µ = 1.53 mm^−^^1^
α = 113.233 (1)°	*T* = 100 K
β = 97.986 (1)°	Plate, brown
γ = 97.339 (1)°	0.51 × 0.15 × 0.08 mm
*V* = 5041.5 (5) Å^3^	

### Data collection

**Table:**

Bruker APEXII DUO CCD area-detector diffractometer	28970 independent reflections
Radiation source: fine-focus sealed tube	21778 reflections with *I* > 2σ(*I*)
graphite	*R*_int_ = 0.033
φ and ω scans	θ_max_ = 30.0°, θ_min_ = 1.9°
Absorption correction: multi-scan (*SADABS*; Bruker, 2009)	*h* = −21→21
*T*_min_ = 0.506, *T*_max_ = 0.885	*k* = −26→26
82542 measured reflections	*l* = −28→28

### Refinement

**Table:**

Refinement on *F*^2^	Primary atom site location: structure-invariant direct methods
Least-squares matrix: full	Secondary atom site location: difference Fourier map
*R*[*F*^2^ > 2σ(*F*^2^)] = 0.045	Hydrogen site location: inferred from neighbouring sites
*wR*(*F*^2^) = 0.121	H-atom parameters constrained
*S* = 1.11	*w* = 1/[σ^2^(*F*_o_^2^) + (0.0352*P*)^2^ + 25.152*P*] where *P* = (*F*_o_^2^ + 2*F*_c_^2^)/3
28970 reflections	(Δ/σ)_max_ = 0.001
1225 parameters	Δρ_max_ = 3.47 e Å^−^^3^
0 restraints	Δρ_min_ = −2.34 e Å^−^^3^

### Special details

**Table:**

Experimental. The crystal was placed in the cold stream of an Oxford Cryosystems Cobra open-flow nitrogen cryostat (Cosier & Glazer, 1986) operating at 100.0 (1) K.
Geometry. All e.s.d.'s (except the e.s.d. in the dihedral angle between two l.s. planes) are estimated using the full covariance matrix. The cell e.s.d.'s are taken into account individually in the estimation of e.s.d.'s in distances, angles and torsion angles; correlations between e.s.d.'s in cell parameters are only used when they are defined by crystal symmetry. An approximate (isotropic) treatment of cell e.s.d.'s is used for estimating e.s.d.'s involving l.s. planes.
Refinement. Refinement of *F*^2^ against ALL reflections. The weighted *R*-factor *wR* and goodness of fit *S* are based on *F*^2^, conventional *R*-factors *R* are based on *F*, with *F* set to zero for negative *F*^2^. The threshold expression of *F*^2^ > σ(*F*^2^) is used only for calculating *R*-factors(gt) *etc*. and is not relevant to the choice of reflections for refinement. *R*-factors based on *F*^2^ are statistically about twice as large as those based on *F*, and *R*- factors based on ALL data will be even larger.

### Fractional atomic coordinates and isotropic or equivalent isotropic displacement parameters (Å^2^)

**Table:**

	*x*	*y*	*z*	*U*_iso_*/*U*_eq_	
Sb1A	−0.061486 (19)	0.701152 (17)	0.978302 (16)	0.02093 (6)	
Ru1A	0.21003 (2)	0.819388 (19)	1.014099 (18)	0.01705 (7)	
Ru2A	0.27903 (2)	0.67570 (2)	0.955200 (18)	0.01778 (7)	
Ru3A	0.09780 (2)	0.66624 (2)	0.973168 (18)	0.01816 (7)	
P1A	0.32886 (8)	0.91401 (6)	1.01639 (6)	0.0191 (2)	
P2A	0.43433 (7)	0.72287 (7)	0.97737 (6)	0.0196 (2)	
O1A	0.1010 (2)	0.8075 (2)	0.8671 (2)	0.0351 (8)	
O2A	0.0940 (2)	0.9232 (2)	1.1051 (2)	0.0333 (8)	
O3A	0.3215 (3)	0.8307 (2)	1.15895 (19)	0.0348 (8)	
O4A	0.3109 (3)	0.6504 (3)	1.0984 (2)	0.0448 (10)	
O5A	0.2593 (3)	0.4989 (2)	0.8596 (3)	0.0493 (11)	
O6A	0.2524 (2)	0.7124 (2)	0.81825 (19)	0.0318 (8)	
O7A	0.0570 (3)	0.6188 (2)	0.8050 (2)	0.0382 (9)	
O8A	0.0677 (3)	0.4919 (2)	0.9450 (2)	0.0409 (9)	
O9A	0.1349 (3)	0.7158 (2)	1.1413 (2)	0.0375 (8)	
C1A	0.3020 (3)	0.9671 (3)	0.9585 (2)	0.0224 (8)	
C2A	0.2874 (4)	0.9255 (3)	0.8817 (3)	0.0299 (10)	
H2AA	0.2923	0.8722	0.8610	0.036*	
C3A	0.2655 (4)	0.9632 (4)	0.8352 (3)	0.0402 (13)	
H3AA	0.2558	0.9350	0.7840	0.048*	
C4A	0.2585 (4)	1.0422 (4)	0.8656 (3)	0.0421 (13)	
H4AA	0.2449	1.0675	0.8348	0.051*	
C5A	0.2714 (4)	1.0842 (3)	0.9420 (3)	0.0377 (12)	
H5AA	0.2655	1.1372	0.9624	0.045*	
C6A	0.2934 (3)	1.0465 (3)	0.9879 (3)	0.0314 (10)	
H6AA	0.3024	1.0748	1.0391	0.038*	
C7A	0.3792 (3)	0.9948 (3)	1.1076 (2)	0.0229 (8)	
C8A	0.3235 (4)	1.0310 (3)	1.1547 (3)	0.0338 (11)	
H8AA	0.2610	1.0119	1.1399	0.041*	
C9A	0.3581 (4)	1.0945 (3)	1.2230 (3)	0.0397 (13)	
H9AA	0.3189	1.1181	1.2529	0.048*	
C10A	0.4501 (4)	1.1227 (3)	1.2466 (3)	0.0388 (12)	
H10A	0.4735	1.1657	1.2923	0.047*	
C11A	0.5076 (4)	1.0868 (3)	1.2017 (3)	0.0376 (12)	
H11A	0.5701	1.1050	1.2180	0.045*	
C12A	0.4734 (3)	1.0240 (3)	1.1330 (3)	0.0299 (10)	
H12A	0.5131	1.0009	1.1033	0.036*	
C13A	0.4276 (3)	0.8756 (3)	0.9828 (2)	0.0227 (8)	
H13A	0.4087	0.8390	0.9308	0.027*	
H13B	0.4729	0.9197	0.9869	0.027*	
C14A	0.4710 (3)	0.8320 (3)	1.0261 (2)	0.0237 (9)	
H14A	0.4548	0.8500	1.0745	0.028*	
H14B	0.5367	0.8459	1.0339	0.028*	
C15A	0.4848 (3)	0.6985 (3)	0.8949 (2)	0.0229 (8)	
C16A	0.4413 (3)	0.6350 (3)	0.8281 (3)	0.0305 (10)	
H16A	0.3863	0.6044	0.8255	0.037*	
C17A	0.4782 (4)	0.6165 (4)	0.7650 (3)	0.0387 (13)	
H17A	0.4489	0.5729	0.7212	0.046*	
C18A	0.5570 (4)	0.6619 (4)	0.7672 (3)	0.0425 (14)	
H18A	0.5808	0.6501	0.7246	0.051*	
C19A	0.6022 (4)	0.7259 (4)	0.8329 (4)	0.0453 (15)	
H19A	0.6562	0.7569	0.8344	0.054*	
C20A	0.5664 (4)	0.7436 (3)	0.8968 (3)	0.0377 (12)	
H20A	0.5975	0.7858	0.9411	0.045*	
C21A	0.5025 (3)	0.6893 (3)	1.0373 (3)	0.0254 (9)	
C22A	0.5174 (5)	0.7281 (4)	1.1142 (3)	0.057 (2)	
H22A	0.4983	0.7760	1.1361	0.069*	
C23A	0.5599 (6)	0.6968 (5)	1.1585 (4)	0.065 (2)	
H23A	0.5673	0.7232	1.2098	0.078*	
C24A	0.5911 (4)	0.6284 (4)	1.1292 (4)	0.0468 (15)	
H24A	0.6210	0.6082	1.1597	0.056*	
C25A	0.5776 (5)	0.5894 (4)	1.0532 (4)	0.0549 (18)	
H25A	0.5980	0.5420	1.0320	0.066*	
C26A	0.5344 (5)	0.6194 (4)	1.0083 (3)	0.0478 (16)	
H26A	0.5264	0.5921	0.9570	0.057*	
C27A	−0.0963 (3)	0.7794 (3)	0.9286 (3)	0.0244 (9)	
C28A	−0.1235 (4)	0.7446 (3)	0.8524 (3)	0.0325 (10)	
H28A	−0.1323	0.6894	0.8272	0.039*	
C29A	−0.1381 (4)	0.7898 (4)	0.8122 (3)	0.0385 (12)	
H29A	−0.1561	0.7650	0.7607	0.046*	
C30A	−0.1256 (4)	0.8714 (4)	0.8493 (3)	0.0399 (13)	
H30A	−0.1354	0.9022	0.8231	0.048*	
C31A	−0.0984 (5)	0.9073 (4)	0.9262 (4)	0.0467 (15)	
H31A	−0.0896	0.9624	0.9513	0.056*	
C32A	−0.0842 (4)	0.8617 (3)	0.9658 (3)	0.0348 (11)	
H32A	−0.0665	0.8863	1.0173	0.042*	
C33A	−0.1068 (3)	0.7415 (3)	1.0806 (3)	0.0264 (9)	
C34A	−0.0888 (4)	0.8221 (3)	1.1319 (3)	0.0374 (12)	
H34A	−0.0580	0.8611	1.1208	0.045*	
C35A	−0.1168 (4)	0.8437 (3)	1.1990 (3)	0.0396 (12)	
H35A	−0.1057	0.8971	1.2323	0.048*	
C36A	−0.1614 (4)	0.7854 (4)	1.2163 (3)	0.0440 (14)	
H36A	−0.1833	0.7997	1.2599	0.053*	
C37A	−0.1725 (5)	0.7078 (4)	1.1692 (3)	0.0508 (17)	
H37A	−0.1978	0.6684	1.1826	0.061*	
C38A	−0.1467 (5)	0.6859 (3)	1.1008 (3)	0.0467 (15)	
H38A	−0.1571	0.6322	1.0685	0.056*	
C39A	−0.1726 (3)	0.6039 (3)	0.9140 (3)	0.0265 (9)	
C40A	−0.1574 (3)	0.5277 (3)	0.8797 (3)	0.0324 (11)	
H40A	−0.0982	0.5189	0.8828	0.039*	
C41A	−0.2300 (4)	0.4641 (3)	0.8404 (3)	0.0388 (12)	
H41A	−0.2197	0.4126	0.8178	0.047*	
C42A	−0.3179 (4)	0.4777 (3)	0.8351 (3)	0.0349 (11)	
H42A	−0.3668	0.4351	0.8094	0.042*	
C43A	−0.3330 (3)	0.5543 (3)	0.8678 (3)	0.0368 (12)	
H43A	−0.3919	0.5634	0.8629	0.044*	
C44A	−0.2608 (3)	0.6175 (3)	0.9078 (3)	0.0349 (11)	
H44A	−0.2712	0.6690	0.9305	0.042*	
C45A	0.2793 (3)	0.8205 (3)	1.1024 (3)	0.0259 (9)	
C46A	0.1361 (3)	0.8842 (3)	1.0680 (2)	0.0226 (8)	
C47A	0.1414 (3)	0.8080 (3)	0.9201 (3)	0.0248 (9)	
C48A	0.2959 (3)	0.6618 (3)	1.0469 (3)	0.0290 (10)	
C49A	0.2688 (3)	0.5651 (3)	0.8966 (3)	0.0300 (10)	
C50A	0.2583 (3)	0.7017 (3)	0.8714 (2)	0.0224 (8)	
C51A	0.0762 (3)	0.6387 (3)	0.8677 (2)	0.0249 (9)	
C52A	0.0763 (3)	0.5579 (3)	0.9553 (3)	0.0267 (9)	
C53A	0.1248 (3)	0.6998 (3)	1.0792 (3)	0.0248 (9)	
Sb1B	0.591162 (19)	0.322816 (17)	0.539002 (15)	0.01948 (6)	
Ru1B	0.32201 (2)	0.19935 (2)	0.497165 (19)	0.02039 (7)	
Ru2B	0.24537 (2)	0.33890 (2)	0.55496 (2)	0.02058 (7)	
Ru3B	0.42965 (2)	0.35418 (2)	0.542128 (18)	0.01812 (7)	
P1B	0.21368 (9)	0.10411 (7)	0.50293 (7)	0.0261 (2)	
P2B	0.09114 (8)	0.28330 (7)	0.52371 (7)	0.0242 (2)	
O1B	0.2092 (2)	0.1901 (2)	0.3535 (2)	0.0332 (8)	
O2B	0.4344 (3)	0.0905 (2)	0.4061 (2)	0.0417 (9)	
O3B	0.4351 (3)	0.2219 (2)	0.6473 (2)	0.0361 (8)	
O4B	0.2727 (3)	0.2988 (2)	0.6902 (2)	0.0365 (8)	
O5B	0.2437 (3)	0.5110 (3)	0.6536 (3)	0.0613 (14)	
O6B	0.2258 (3)	0.3753 (3)	0.4175 (3)	0.0490 (11)	
O7B	0.3918 (2)	0.2976 (2)	0.37254 (19)	0.0339 (8)	
O8B	0.4642 (3)	0.5285 (2)	0.5714 (2)	0.0411 (9)	
O9B	0.4645 (3)	0.4055 (2)	0.7112 (2)	0.0364 (8)	
C1B	0.1669 (3)	0.0142 (3)	0.4166 (3)	0.0310 (10)	
C2B	0.1947 (3)	0.0015 (3)	0.3512 (3)	0.0315 (10)	
H2BA	0.2379	0.0408	0.3497	0.038*	
C3B	0.1604 (4)	−0.0680 (3)	0.2873 (3)	0.0397 (12)	
H3BA	0.1809	−0.0752	0.2439	0.048*	
C4B	0.0965 (4)	−0.1258 (4)	0.2885 (3)	0.0475 (15)	
H4BA	0.0719	−0.1719	0.2454	0.057*	
C5B	0.0682 (4)	−0.1159 (4)	0.3538 (4)	0.0485 (15)	
H5BA	0.0253	−0.1558	0.3548	0.058*	
C6B	0.1035 (4)	−0.0470 (3)	0.4175 (3)	0.0403 (13)	
H6BA	0.0851	−0.0411	0.4614	0.048*	
C7B	0.2515 (4)	0.0621 (3)	0.5682 (3)	0.0304 (10)	
C8B	0.3394 (4)	0.0449 (3)	0.5725 (3)	0.0384 (12)	
H8BA	0.3764	0.0556	0.5428	0.046*	
C9B	0.3718 (5)	0.0124 (4)	0.6201 (3)	0.0467 (15)	
H9BA	0.4302	0.0018	0.6226	0.056*	
C10B	0.3171 (6)	−0.0041 (4)	0.6639 (4)	0.0557 (18)	
H10B	0.3389	−0.0256	0.6962	0.067*	
C11B	0.2304 (5)	0.0109 (5)	0.6599 (4)	0.0569 (18)	
H11B	0.1933	−0.0019	0.6885	0.068*	
C12B	0.1974 (5)	0.0454 (4)	0.6130 (3)	0.0447 (14)	
H12B	0.1395	0.0570	0.6119	0.054*	
C13B	0.1080 (4)	0.1364 (3)	0.5292 (3)	0.0325 (11)	
H13C	0.0655	0.0907	0.5255	0.039*	
H13D	0.1222	0.1753	0.5805	0.039*	
C14B	0.0626 (3)	0.1730 (3)	0.4805 (3)	0.0318 (10)	
H14C	−0.0028	0.1556	0.4707	0.038*	
H14D	0.0816	0.1534	0.4332	0.038*	
C15B	0.0242 (3)	0.3097 (3)	0.4569 (3)	0.0284 (10)	
C16B	−0.0167 (4)	0.3756 (3)	0.4824 (3)	0.0365 (11)	
H16B	−0.0133	0.4030	0.5331	0.044*	
C17B	−0.0626 (4)	0.4012 (4)	0.4335 (3)	0.0442 (14)	
H17B	−0.0895	0.4454	0.4515	0.053*	
C18B	−0.0681 (4)	0.3604 (4)	0.3581 (3)	0.0451 (15)	
H18B	−0.0993	0.3768	0.3250	0.054*	
C19B	−0.0273 (4)	0.2955 (4)	0.3321 (3)	0.0454 (15)	
H19B	−0.0301	0.2687	0.2814	0.054*	
C20B	0.0180 (4)	0.2699 (4)	0.3809 (3)	0.0406 (13)	
H20B	0.0445	0.2255	0.3625	0.049*	
C21B	0.0274 (3)	0.3058 (3)	0.5985 (3)	0.0269 (9)	
C22B	0.0680 (4)	0.3562 (4)	0.6709 (3)	0.0430 (14)	
H22B	0.1290	0.3806	0.6827	0.052*	
C23B	0.0178 (5)	0.3708 (5)	0.7267 (4)	0.059 (2)	
H23B	0.0457	0.4051	0.7755	0.071*	
C24B	−0.0712 (5)	0.3355 (5)	0.7106 (4)	0.0568 (19)	
H24B	−0.1037	0.3446	0.7483	0.068*	
C25B	−0.1140 (5)	0.2854 (5)	0.6371 (4)	0.0548 (18)	
H25B	−0.1753	0.2620	0.6256	0.066*	
C26B	−0.0646 (4)	0.2708 (4)	0.5815 (3)	0.0400 (13)	
H26B	−0.0930	0.2375	0.5326	0.048*	
C27B	0.6993 (3)	0.4228 (3)	0.6026 (2)	0.0227 (8)	
C28B	0.7858 (4)	0.4236 (3)	0.5888 (4)	0.0427 (14)	
H28B	0.7972	0.3809	0.5494	0.051*	
C29B	0.8555 (4)	0.4873 (3)	0.6331 (4)	0.0478 (16)	
H29B	0.9138	0.4867	0.6236	0.057*	
C30B	0.8404 (3)	0.5514 (3)	0.6907 (3)	0.0318 (10)	
H30B	0.8879	0.5940	0.7203	0.038*	
C31B	0.7536 (4)	0.5520 (3)	0.7043 (3)	0.0375 (12)	
H31B	0.7424	0.5954	0.7432	0.045*	
C32B	0.6835 (4)	0.4885 (3)	0.6605 (3)	0.0349 (11)	
H32B	0.6251	0.4896	0.6697	0.042*	
C33B	0.6441 (3)	0.2765 (3)	0.4407 (2)	0.0217 (8)	
C34B	0.6234 (3)	0.3025 (3)	0.3861 (3)	0.0263 (9)	
H34B	0.5800	0.3342	0.3894	0.032*	
C35B	0.6664 (4)	0.2822 (3)	0.3259 (3)	0.0313 (10)	
H35B	0.6512	0.2995	0.2889	0.038*	
C36B	0.7323 (3)	0.2358 (3)	0.3216 (3)	0.0289 (10)	
H36B	0.7621	0.2228	0.2821	0.035*	
C37B	0.7536 (3)	0.2093 (3)	0.3758 (3)	0.0327 (11)	
H37B	0.7972	0.1778	0.3724	0.039*	
C38B	0.7100 (3)	0.2292 (3)	0.4359 (3)	0.0320 (11)	
H38B	0.7247	0.2112	0.4724	0.038*	
C39B	0.6245 (3)	0.2472 (3)	0.5910 (2)	0.0224 (8)	
C40B	0.6090 (4)	0.1649 (3)	0.5533 (3)	0.0318 (10)	
H40B	0.5897	0.1410	0.5019	0.038*	
C41B	0.6220 (5)	0.1176 (3)	0.5918 (3)	0.0429 (14)	
H41B	0.6121	0.0624	0.5661	0.052*	
C42B	0.6495 (4)	0.1528 (4)	0.6680 (3)	0.0420 (13)	
H42B	0.6568	0.1211	0.6937	0.050*	
C43B	0.6664 (4)	0.2347 (3)	0.7063 (3)	0.0345 (11)	
H43B	0.6859	0.2582	0.7577	0.041*	
C44B	0.6541 (3)	0.2822 (3)	0.6682 (3)	0.0272 (9)	
H44B	0.6656	0.3375	0.6941	0.033*	
C45B	0.2505 (3)	0.1991 (3)	0.4099 (3)	0.0248 (9)	
C46B	0.3946 (3)	0.1328 (3)	0.4428 (2)	0.0265 (9)	
C47B	0.3945 (3)	0.2181 (3)	0.5931 (3)	0.0265 (9)	
C48B	0.2661 (3)	0.3111 (3)	0.6383 (3)	0.0269 (9)	
C49B	0.2434 (4)	0.4467 (3)	0.6153 (3)	0.0353 (11)	
C50B	0.2353 (3)	0.3600 (3)	0.4666 (3)	0.0323 (11)	
C51B	0.4029 (3)	0.3167 (3)	0.4355 (3)	0.0246 (9)	
C52B	0.4522 (3)	0.4623 (3)	0.5594 (3)	0.0262 (9)	
C53B	0.4480 (3)	0.3832 (3)	0.6477 (3)	0.0251 (9)	

### Atomic displacement parameters (Å^2^)

**Table:**

	*U*^11^	*U*^22^	*U*^33^	*U*^12^	*U*^13^	*U*^23^
Sb1A	0.01861 (13)	0.02543 (14)	0.02117 (13)	0.00765 (11)	0.00624 (10)	0.01061 (11)
Ru1A	0.01765 (15)	0.01983 (15)	0.01511 (14)	0.00562 (12)	0.00476 (12)	0.00779 (12)
Ru2A	0.01684 (15)	0.02065 (15)	0.01764 (15)	0.00657 (12)	0.00564 (12)	0.00836 (12)
Ru3A	0.01704 (15)	0.02083 (15)	0.01852 (15)	0.00543 (12)	0.00573 (12)	0.00904 (12)
P1A	0.0210 (5)	0.0193 (5)	0.0165 (5)	0.0035 (4)	0.0053 (4)	0.0067 (4)
P2A	0.0170 (5)	0.0247 (5)	0.0172 (5)	0.0063 (4)	0.0044 (4)	0.0080 (4)
O1A	0.0322 (19)	0.047 (2)	0.0255 (17)	0.0062 (16)	0.0008 (14)	0.0161 (16)
O2A	0.0356 (19)	0.0302 (17)	0.0361 (19)	0.0141 (15)	0.0184 (16)	0.0101 (15)
O3A	0.041 (2)	0.043 (2)	0.0249 (17)	0.0106 (17)	0.0008 (15)	0.0198 (16)
O4A	0.055 (3)	0.063 (3)	0.039 (2)	0.028 (2)	0.0200 (19)	0.037 (2)
O5A	0.052 (3)	0.0236 (18)	0.061 (3)	0.0103 (18)	0.016 (2)	0.0041 (18)
O6A	0.0326 (19)	0.046 (2)	0.0236 (16)	0.0126 (16)	0.0102 (14)	0.0181 (15)
O7A	0.0311 (19)	0.054 (2)	0.0235 (17)	−0.0002 (17)	0.0037 (15)	0.0135 (17)
O8A	0.045 (2)	0.0284 (18)	0.056 (3)	0.0123 (17)	0.0181 (19)	0.0205 (18)
O9A	0.041 (2)	0.051 (2)	0.0263 (18)	0.0113 (18)	0.0122 (16)	0.0196 (17)
C1A	0.020 (2)	0.026 (2)	0.024 (2)	0.0015 (16)	0.0035 (16)	0.0140 (17)
C2A	0.037 (3)	0.032 (2)	0.023 (2)	0.008 (2)	0.0072 (19)	0.0124 (19)
C3A	0.049 (3)	0.052 (3)	0.024 (2)	0.016 (3)	0.007 (2)	0.020 (2)
C4A	0.044 (3)	0.054 (3)	0.044 (3)	0.017 (3)	0.008 (3)	0.035 (3)
C5A	0.039 (3)	0.034 (3)	0.044 (3)	0.008 (2)	0.002 (2)	0.022 (2)
C6A	0.033 (3)	0.033 (2)	0.028 (2)	0.005 (2)	0.001 (2)	0.014 (2)
C7A	0.023 (2)	0.023 (2)	0.0193 (19)	−0.0009 (16)	0.0017 (16)	0.0076 (16)
C8A	0.029 (2)	0.033 (3)	0.027 (2)	0.001 (2)	0.007 (2)	0.001 (2)
C9A	0.038 (3)	0.037 (3)	0.030 (3)	0.004 (2)	0.009 (2)	−0.001 (2)
C10A	0.042 (3)	0.031 (3)	0.026 (2)	−0.003 (2)	0.001 (2)	0.000 (2)
C11A	0.031 (3)	0.033 (3)	0.034 (3)	−0.004 (2)	−0.001 (2)	0.006 (2)
C12A	0.026 (2)	0.028 (2)	0.029 (2)	0.0025 (19)	0.0050 (19)	0.0064 (19)
C13A	0.023 (2)	0.023 (2)	0.021 (2)	0.0033 (16)	0.0059 (16)	0.0076 (16)
C14A	0.021 (2)	0.028 (2)	0.0194 (19)	0.0053 (17)	0.0022 (16)	0.0078 (17)
C15A	0.022 (2)	0.028 (2)	0.024 (2)	0.0109 (17)	0.0079 (17)	0.0135 (18)
C16A	0.027 (2)	0.041 (3)	0.023 (2)	0.012 (2)	0.0096 (18)	0.010 (2)
C17A	0.042 (3)	0.057 (3)	0.025 (2)	0.030 (3)	0.015 (2)	0.017 (2)
C18A	0.061 (4)	0.053 (3)	0.042 (3)	0.036 (3)	0.036 (3)	0.033 (3)
C19A	0.046 (3)	0.043 (3)	0.063 (4)	0.014 (3)	0.037 (3)	0.029 (3)
C20A	0.040 (3)	0.031 (3)	0.045 (3)	0.007 (2)	0.022 (2)	0.014 (2)
C21A	0.019 (2)	0.036 (2)	0.025 (2)	0.0095 (18)	0.0063 (17)	0.0139 (19)
C22A	0.084 (5)	0.064 (4)	0.023 (3)	0.047 (4)	0.002 (3)	0.010 (3)
C23A	0.087 (6)	0.084 (5)	0.026 (3)	0.047 (5)	0.001 (3)	0.021 (3)
C24A	0.048 (4)	0.063 (4)	0.045 (3)	0.024 (3)	0.007 (3)	0.036 (3)
C25A	0.079 (5)	0.056 (4)	0.041 (3)	0.039 (4)	0.009 (3)	0.026 (3)
C26A	0.071 (4)	0.050 (3)	0.031 (3)	0.036 (3)	0.010 (3)	0.018 (3)
C27A	0.019 (2)	0.033 (2)	0.025 (2)	0.0105 (18)	0.0057 (17)	0.0157 (19)
C28A	0.038 (3)	0.032 (2)	0.027 (2)	0.006 (2)	0.006 (2)	0.013 (2)
C29A	0.043 (3)	0.050 (3)	0.028 (3)	0.012 (3)	0.008 (2)	0.021 (2)
C30A	0.045 (3)	0.048 (3)	0.041 (3)	0.021 (3)	0.008 (2)	0.030 (3)
C31A	0.064 (4)	0.034 (3)	0.045 (3)	0.020 (3)	0.006 (3)	0.018 (3)
C32A	0.044 (3)	0.036 (3)	0.029 (2)	0.018 (2)	0.005 (2)	0.015 (2)
C33A	0.028 (2)	0.034 (2)	0.025 (2)	0.0155 (19)	0.0118 (18)	0.0158 (19)
C34A	0.041 (3)	0.032 (3)	0.036 (3)	0.004 (2)	0.012 (2)	0.011 (2)
C35A	0.043 (3)	0.038 (3)	0.032 (3)	0.007 (2)	0.010 (2)	0.009 (2)
C36A	0.051 (4)	0.052 (3)	0.033 (3)	0.010 (3)	0.021 (3)	0.017 (3)
C37A	0.083 (5)	0.038 (3)	0.043 (3)	0.008 (3)	0.033 (3)	0.024 (3)
C38A	0.078 (5)	0.031 (3)	0.039 (3)	0.014 (3)	0.027 (3)	0.016 (2)
C39A	0.026 (2)	0.035 (2)	0.026 (2)	0.0124 (19)	0.0092 (18)	0.0179 (19)
C40A	0.022 (2)	0.034 (3)	0.039 (3)	0.0036 (19)	0.003 (2)	0.014 (2)
C41A	0.031 (3)	0.030 (3)	0.044 (3)	0.004 (2)	0.007 (2)	0.006 (2)
C42A	0.027 (2)	0.040 (3)	0.032 (3)	−0.002 (2)	0.001 (2)	0.015 (2)
C43A	0.019 (2)	0.050 (3)	0.042 (3)	0.005 (2)	0.004 (2)	0.021 (3)
C44A	0.023 (2)	0.041 (3)	0.040 (3)	0.011 (2)	0.004 (2)	0.015 (2)
C45A	0.030 (2)	0.028 (2)	0.026 (2)	0.0105 (19)	0.0085 (18)	0.0158 (19)
C46A	0.022 (2)	0.026 (2)	0.024 (2)	0.0044 (17)	0.0086 (17)	0.0143 (17)
C47A	0.022 (2)	0.029 (2)	0.023 (2)	0.0057 (17)	0.0050 (17)	0.0096 (18)
C48A	0.033 (3)	0.033 (2)	0.030 (2)	0.015 (2)	0.014 (2)	0.018 (2)
C49A	0.025 (2)	0.029 (2)	0.036 (3)	0.0091 (19)	0.009 (2)	0.013 (2)
C50A	0.018 (2)	0.026 (2)	0.022 (2)	0.0044 (16)	0.0044 (16)	0.0094 (17)
C51A	0.022 (2)	0.027 (2)	0.022 (2)	−0.0015 (17)	0.0039 (17)	0.0084 (17)
C52A	0.024 (2)	0.029 (2)	0.028 (2)	0.0078 (18)	0.0090 (18)	0.0116 (19)
C53A	0.023 (2)	0.028 (2)	0.024 (2)	0.0048 (17)	0.0056 (17)	0.0113 (18)
Sb1B	0.01926 (13)	0.02293 (13)	0.01749 (12)	0.00678 (10)	0.00551 (10)	0.00843 (10)
Ru1B	0.02591 (17)	0.02054 (15)	0.01923 (15)	0.00768 (13)	0.00992 (13)	0.01034 (13)
Ru2B	0.02011 (16)	0.02150 (16)	0.02397 (17)	0.00693 (13)	0.01028 (13)	0.01081 (13)
Ru3B	0.01884 (16)	0.02118 (15)	0.01860 (15)	0.00665 (12)	0.00762 (12)	0.01070 (12)
P1B	0.0347 (6)	0.0212 (5)	0.0257 (6)	0.0054 (5)	0.0161 (5)	0.0101 (5)
P2B	0.0210 (5)	0.0293 (6)	0.0229 (5)	0.0038 (4)	0.0088 (4)	0.0106 (5)
O1B	0.0322 (19)	0.045 (2)	0.0300 (18)	0.0109 (16)	0.0067 (15)	0.0222 (16)
O2B	0.038 (2)	0.051 (2)	0.033 (2)	0.0233 (18)	0.0120 (17)	0.0094 (18)
O3B	0.047 (2)	0.0373 (19)	0.0271 (18)	0.0160 (17)	0.0082 (16)	0.0141 (16)
O4B	0.039 (2)	0.050 (2)	0.0294 (18)	0.0110 (17)	0.0159 (16)	0.0224 (17)
O5B	0.064 (3)	0.030 (2)	0.077 (4)	0.016 (2)	0.020 (3)	0.005 (2)
O6B	0.037 (2)	0.079 (3)	0.062 (3)	0.024 (2)	0.018 (2)	0.056 (3)
O7B	0.0318 (19)	0.047 (2)	0.0248 (17)	0.0002 (16)	0.0077 (14)	0.0189 (16)
O8B	0.068 (3)	0.0246 (17)	0.040 (2)	0.0158 (18)	0.027 (2)	0.0158 (16)
O9B	0.039 (2)	0.046 (2)	0.0244 (17)	0.0063 (17)	0.0077 (15)	0.0146 (16)
C1B	0.030 (2)	0.027 (2)	0.032 (3)	0.0026 (19)	0.012 (2)	0.008 (2)
C2B	0.030 (2)	0.035 (3)	0.030 (2)	0.007 (2)	0.008 (2)	0.013 (2)
C3B	0.041 (3)	0.041 (3)	0.030 (3)	0.011 (2)	0.011 (2)	0.006 (2)
C4B	0.037 (3)	0.039 (3)	0.042 (3)	0.001 (2)	0.003 (2)	−0.005 (3)
C5B	0.044 (3)	0.036 (3)	0.048 (4)	−0.002 (3)	0.006 (3)	0.004 (3)
C6B	0.043 (3)	0.033 (3)	0.038 (3)	−0.001 (2)	0.014 (2)	0.009 (2)
C7B	0.044 (3)	0.024 (2)	0.026 (2)	0.007 (2)	0.013 (2)	0.0115 (19)
C8B	0.047 (3)	0.034 (3)	0.038 (3)	0.014 (2)	0.013 (2)	0.016 (2)
C9B	0.067 (4)	0.041 (3)	0.039 (3)	0.028 (3)	0.010 (3)	0.019 (3)
C10B	0.086 (5)	0.054 (4)	0.050 (4)	0.033 (4)	0.026 (4)	0.035 (3)
C11B	0.069 (5)	0.081 (5)	0.047 (4)	0.023 (4)	0.026 (3)	0.047 (4)
C12B	0.054 (4)	0.049 (3)	0.043 (3)	0.018 (3)	0.026 (3)	0.024 (3)
C13B	0.038 (3)	0.025 (2)	0.037 (3)	0.004 (2)	0.022 (2)	0.012 (2)
C14B	0.026 (2)	0.031 (2)	0.031 (2)	−0.0012 (19)	0.0082 (19)	0.006 (2)
C15B	0.022 (2)	0.038 (3)	0.024 (2)	0.0003 (19)	0.0054 (17)	0.013 (2)
C16B	0.038 (3)	0.037 (3)	0.031 (3)	0.001 (2)	0.000 (2)	0.015 (2)
C17B	0.050 (4)	0.038 (3)	0.046 (3)	0.004 (3)	0.001 (3)	0.024 (3)
C18B	0.033 (3)	0.063 (4)	0.040 (3)	−0.013 (3)	−0.006 (2)	0.033 (3)
C19B	0.029 (3)	0.076 (4)	0.028 (3)	0.001 (3)	0.002 (2)	0.022 (3)
C20B	0.027 (3)	0.065 (4)	0.028 (3)	0.011 (3)	0.008 (2)	0.018 (3)
C21B	0.025 (2)	0.039 (3)	0.028 (2)	0.0136 (19)	0.0134 (18)	0.021 (2)
C22B	0.030 (3)	0.066 (4)	0.031 (3)	0.016 (3)	0.013 (2)	0.014 (3)
C23B	0.056 (4)	0.096 (6)	0.032 (3)	0.040 (4)	0.022 (3)	0.022 (3)
C24B	0.056 (4)	0.089 (5)	0.060 (4)	0.044 (4)	0.045 (4)	0.047 (4)
C25B	0.040 (3)	0.073 (5)	0.069 (5)	0.021 (3)	0.036 (3)	0.037 (4)
C26B	0.031 (3)	0.052 (3)	0.044 (3)	0.013 (2)	0.017 (2)	0.022 (3)
C27B	0.0159 (19)	0.030 (2)	0.023 (2)	0.0048 (16)	0.0004 (16)	0.0130 (18)
C28B	0.027 (3)	0.033 (3)	0.054 (4)	0.008 (2)	0.010 (2)	0.003 (2)
C29B	0.023 (3)	0.037 (3)	0.067 (4)	0.007 (2)	0.012 (3)	0.005 (3)
C30B	0.024 (2)	0.034 (3)	0.031 (2)	−0.0051 (19)	−0.0015 (19)	0.012 (2)
C31B	0.036 (3)	0.037 (3)	0.027 (2)	−0.002 (2)	0.008 (2)	0.002 (2)
C32B	0.032 (3)	0.036 (3)	0.029 (2)	0.002 (2)	0.012 (2)	0.005 (2)
C33B	0.021 (2)	0.025 (2)	0.0202 (19)	0.0062 (16)	0.0057 (16)	0.0090 (16)
C34B	0.027 (2)	0.025 (2)	0.028 (2)	0.0061 (18)	0.0092 (18)	0.0113 (18)
C35B	0.034 (3)	0.039 (3)	0.024 (2)	0.007 (2)	0.012 (2)	0.016 (2)
C36B	0.028 (2)	0.033 (2)	0.021 (2)	0.0016 (19)	0.0107 (18)	0.0058 (18)
C37B	0.028 (2)	0.040 (3)	0.031 (2)	0.016 (2)	0.011 (2)	0.012 (2)
C38B	0.031 (3)	0.046 (3)	0.027 (2)	0.019 (2)	0.012 (2)	0.018 (2)
C39B	0.019 (2)	0.030 (2)	0.021 (2)	0.0092 (17)	0.0039 (16)	0.0125 (17)
C40B	0.038 (3)	0.034 (2)	0.023 (2)	0.013 (2)	0.006 (2)	0.0096 (19)
C41B	0.070 (4)	0.027 (2)	0.037 (3)	0.022 (3)	0.016 (3)	0.014 (2)
C42B	0.054 (4)	0.050 (3)	0.042 (3)	0.026 (3)	0.013 (3)	0.034 (3)
C43B	0.036 (3)	0.045 (3)	0.027 (2)	0.009 (2)	0.004 (2)	0.020 (2)
C44B	0.025 (2)	0.030 (2)	0.023 (2)	0.0042 (18)	0.0038 (17)	0.0078 (18)
C45B	0.021 (2)	0.027 (2)	0.033 (2)	0.0066 (17)	0.0129 (18)	0.0172 (19)
C46B	0.032 (2)	0.031 (2)	0.020 (2)	0.0111 (19)	0.0082 (18)	0.0121 (18)
C47B	0.033 (2)	0.027 (2)	0.023 (2)	0.0099 (19)	0.0088 (18)	0.0110 (18)
C48B	0.027 (2)	0.028 (2)	0.025 (2)	0.0034 (18)	0.0106 (18)	0.0097 (18)
C49B	0.027 (2)	0.031 (3)	0.046 (3)	0.011 (2)	0.011 (2)	0.012 (2)
C50B	0.024 (2)	0.042 (3)	0.046 (3)	0.014 (2)	0.015 (2)	0.029 (2)
C51B	0.021 (2)	0.034 (2)	0.026 (2)	0.0069 (18)	0.0094 (17)	0.0168 (19)
C52B	0.036 (3)	0.028 (2)	0.022 (2)	0.0137 (19)	0.0150 (19)	0.0130 (18)
C53B	0.025 (2)	0.026 (2)	0.027 (2)	0.0069 (18)	0.0097 (18)	0.0125 (18)

### Geometric parameters (Å, °)

**Table:**

Sb1A—C33A	2.133 (4)	Sb1B—C39B	2.125 (4)
Sb1A—C39A	2.133 (5)	Sb1B—C27B	2.132 (4)
Sb1A—C27A	2.142 (5)	Sb1B—C33B	2.132 (4)
Sb1A—Ru3A	2.5846 (4)	Sb1B—Ru3B	2.5919 (4)
Ru1A—C46A	1.879 (4)	Ru1B—C46B	1.884 (4)
Ru1A—C45A	1.919 (5)	Ru1B—C45B	1.923 (5)
Ru1A—C47A	1.939 (5)	Ru1B—C47B	1.953 (5)
Ru1A—P1A	2.3350 (12)	Ru1B—P1B	2.3073 (12)
Ru1A—Ru2A	2.8651 (5)	Ru1B—Ru3B	2.8500 (5)
Ru1A—Ru3A	2.8676 (5)	Ru1B—Ru2B	2.8655 (5)
Ru2A—C49A	1.898 (5)	Ru2B—C49B	1.898 (5)
Ru2A—C50A	1.919 (5)	Ru2B—C48B	1.932 (5)
Ru2A—C48A	1.942 (5)	Ru2B—C50B	1.950 (5)
Ru2A—P2A	2.3202 (12)	Ru2B—P2B	2.3224 (12)
Ru2A—Ru3A	2.8124 (5)	Ru2B—Ru3B	2.8292 (5)
Ru3A—C52A	1.880 (5)	Ru3B—C52B	1.879 (5)
Ru3A—C53A	1.929 (5)	Ru3B—C51B	1.930 (5)
Ru3A—C51A	1.936 (5)	Ru3B—C53B	1.935 (5)
P1A—C7A	1.826 (4)	P1B—C7B	1.834 (5)
P1A—C1A	1.835 (5)	P1B—C1B	1.836 (5)
P1A—C13A	1.838 (4)	P1B—C13B	1.853 (5)
P2A—C21A	1.820 (5)	P2B—C15B	1.826 (5)
P2A—C15A	1.836 (4)	P2B—C21B	1.834 (4)
P2A—C14A	1.839 (5)	P2B—C14B	1.850 (5)
O1A—C47A	1.146 (6)	O1B—C45B	1.151 (6)
O2A—C46A	1.145 (5)	O2B—C46B	1.146 (6)
O3A—C45A	1.152 (6)	O3B—C47B	1.143 (6)
O4A—C48A	1.131 (6)	O4B—C48B	1.145 (6)
O5A—C49A	1.137 (6)	O5B—C49B	1.137 (6)
O6A—C50A	1.153 (5)	O6B—C50B	1.125 (6)
O7A—C51A	1.142 (6)	O7B—C51B	1.149 (5)
O8A—C52A	1.148 (6)	O8B—C52B	1.143 (6)
O9A—C53A	1.142 (6)	O9B—C53B	1.149 (6)
C1A—C6A	1.389 (7)	C1B—C2B	1.374 (7)
C1A—C2A	1.393 (6)	C1B—C6B	1.401 (7)
C2A—C3A	1.399 (7)	C2B—C3B	1.385 (7)
C2A—H2AA	0.9300	C2B—H2BA	0.9300
C3A—C4A	1.376 (8)	C3B—C4B	1.363 (9)
C3A—H3AA	0.9300	C3B—H3BA	0.9300
C4A—C5A	1.388 (8)	C4B—C5B	1.386 (9)
C4A—H4AA	0.9300	C4B—H4BA	0.9300
C5A—C6A	1.391 (7)	C5B—C6B	1.380 (8)
C5A—H5AA	0.9300	C5B—H5BA	0.9300
C6A—H6AA	0.9300	C6B—H6BA	0.9300
C7A—C8A	1.386 (6)	C7B—C12B	1.390 (7)
C7A—C12A	1.406 (6)	C7B—C8B	1.409 (8)
C8A—C9A	1.383 (7)	C8B—C9B	1.386 (8)
C8A—H8AA	0.9300	C8B—H8BA	0.9300
C9A—C10A	1.370 (8)	C9B—C10B	1.379 (9)
C9A—H9AA	0.9300	C9B—H9BA	0.9300
C10A—C11A	1.381 (8)	C10B—C11B	1.377 (10)
C10A—H10A	0.9300	C10B—H10B	0.9300
C11A—C12A	1.381 (7)	C11B—C12B	1.405 (9)
C11A—H11A	0.9300	C11B—H11B	0.9300
C12A—H12A	0.9300	C12B—H12B	0.9300
C13A—C14A	1.542 (6)	C13B—C14B	1.532 (8)
C13A—H13A	0.9700	C13B—H13C	0.9700
C13A—H13B	0.9700	C13B—H13D	0.9700
C14A—H14A	0.9700	C14B—H14C	0.9700
C14A—H14B	0.9700	C14B—H14D	0.9700
C15A—C20A	1.390 (7)	C15B—C20B	1.390 (7)
C15A—C16A	1.390 (7)	C15B—C16B	1.391 (8)
C16A—C17A	1.390 (6)	C16B—C17B	1.390 (8)
C16A—H16A	0.9300	C16B—H16B	0.9300
C17A—C18A	1.357 (9)	C17B—C18B	1.383 (9)
C17A—H17A	0.9300	C17B—H17B	0.9300
C18A—C19A	1.387 (9)	C18B—C19B	1.378 (10)
C18A—H18A	0.9300	C18B—H18B	0.9300
C19A—C20A	1.396 (7)	C19B—C20B	1.385 (8)
C19A—H19A	0.9300	C19B—H19B	0.9300
C20A—H20A	0.9300	C20B—H20B	0.9300
C21A—C26A	1.381 (7)	C21B—C22B	1.376 (7)
C21A—C22A	1.390 (7)	C21B—C26B	1.395 (7)
C22A—C23A	1.373 (8)	C22B—C23B	1.398 (8)
C22A—H22A	0.9300	C22B—H22B	0.9300
C23A—C24A	1.355 (9)	C23B—C24B	1.357 (10)
C23A—H23A	0.9300	C23B—H23B	0.9300
C24A—C25A	1.376 (9)	C24B—C25B	1.398 (11)
C24A—H24A	0.9300	C24B—H24B	0.9300
C25A—C26A	1.370 (8)	C25B—C26B	1.386 (8)
C25A—H25A	0.9300	C25B—H25B	0.9300
C26A—H26A	0.9300	C26B—H26B	0.9300
C27A—C28A	1.376 (7)	C27B—C28B	1.376 (7)
C27A—C32A	1.388 (7)	C27B—C32B	1.389 (6)
C28A—C29A	1.393 (7)	C28B—C29B	1.379 (8)
C28A—H28A	0.9300	C28B—H28B	0.9300
C29A—C30A	1.376 (8)	C29B—C30B	1.364 (8)
C29A—H29A	0.9300	C29B—H29B	0.9300
C30A—C31A	1.389 (8)	C30B—C31B	1.382 (7)
C30A—H30A	0.9300	C30B—H30B	0.9300
C31A—C32A	1.389 (8)	C31B—C32B	1.377 (7)
C31A—H31A	0.9300	C31B—H31B	0.9300
C32A—H32A	0.9300	C32B—H32B	0.9300
C33A—C38A	1.357 (7)	C33B—C34B	1.374 (6)
C33A—C34A	1.408 (7)	C33B—C38B	1.403 (6)
C34A—C35A	1.387 (7)	C34B—C35B	1.395 (6)
C34A—H34A	0.9300	C34B—H34B	0.9300
C35A—C36A	1.390 (8)	C35B—C36B	1.391 (7)
C35A—H35A	0.9300	C35B—H35B	0.9300
C36A—C37A	1.350 (8)	C36B—C37B	1.377 (7)
C36A—H36A	0.9300	C36B—H36B	0.9300
C37A—C38A	1.396 (8)	C37B—C38B	1.398 (6)
C37A—H37A	0.9300	C37B—H37B	0.9300
C38A—H38A	0.9300	C38B—H38B	0.9300
C39A—C40A	1.376 (7)	C39B—C40B	1.385 (7)
C39A—C44A	1.390 (6)	C39B—C44B	1.398 (6)
C40A—C41A	1.387 (7)	C40B—C41B	1.396 (7)
C40A—H40A	0.9300	C40B—H40B	0.9300
C41A—C42A	1.384 (7)	C41B—C42B	1.378 (8)
C41A—H41A	0.9300	C41B—H41B	0.9300
C42A—C43A	1.379 (8)	C42B—C43B	1.379 (8)
C42A—H42A	0.9300	C42B—H42B	0.9300
C43A—C44A	1.381 (8)	C43B—C44B	1.392 (7)
C43A—H43A	0.9300	C43B—H43B	0.9300
C44A—H44A	0.9300	C44B—H44B	0.9300
			
C33A—Sb1A—C39A	98.08 (19)	C39B—Sb1B—C27B	98.51 (17)
C33A—Sb1A—C27A	105.68 (17)	C39B—Sb1B—C33B	102.13 (16)
C39A—Sb1A—C27A	97.20 (18)	C27B—Sb1B—C33B	97.89 (17)
C33A—Sb1A—Ru3A	120.39 (13)	C39B—Sb1B—Ru3B	115.01 (12)
C39A—Sb1A—Ru3A	115.03 (12)	C27B—Sb1B—Ru3B	114.69 (12)
C27A—Sb1A—Ru3A	116.63 (12)	C33B—Sb1B—Ru3B	124.42 (12)
C46A—Ru1A—C45A	92.10 (19)	C46B—Ru1B—C45B	93.28 (19)
C46A—Ru1A—C47A	92.31 (19)	C46B—Ru1B—C47B	93.58 (19)
C45A—Ru1A—C47A	174.59 (19)	C45B—Ru1B—C47B	170.68 (19)
C46A—Ru1A—P1A	101.91 (14)	C46B—Ru1B—P1B	99.49 (15)
C45A—Ru1A—P1A	88.87 (15)	C45B—Ru1B—P1B	93.42 (14)
C47A—Ru1A—P1A	93.28 (14)	C47B—Ru1B—P1B	91.67 (15)
C46A—Ru1A—Ru2A	154.44 (13)	C46B—Ru1B—Ru3B	102.02 (15)
C45A—Ru1A—Ru2A	78.44 (14)	C45B—Ru1B—Ru3B	94.56 (14)
C47A—Ru1A—Ru2A	96.26 (13)	C47B—Ru1B—Ru3B	77.83 (14)
P1A—Ru1A—Ru2A	101.59 (3)	P1B—Ru1B—Ru3B	156.56 (3)
C46A—Ru1A—Ru3A	98.87 (14)	C46B—Ru1B—Ru2B	157.28 (15)
C45A—Ru1A—Ru3A	93.91 (15)	C45B—Ru1B—Ru2B	76.72 (13)
C47A—Ru1A—Ru3A	82.31 (14)	C47B—Ru1B—Ru2B	94.62 (13)
P1A—Ru1A—Ru3A	158.92 (3)	P1B—Ru1B—Ru2B	101.41 (3)
Ru2A—Ru1A—Ru3A	58.759 (12)	Ru3B—Ru1B—Ru2B	59.339 (12)
C49A—Ru2A—C50A	94.3 (2)	C49B—Ru2B—C48B	93.7 (2)
C49A—Ru2A—C48A	92.3 (2)	C49B—Ru2B—C50B	91.1 (2)
C50A—Ru2A—C48A	172.99 (19)	C48B—Ru2B—C50B	173.32 (19)
C49A—Ru2A—P2A	101.77 (15)	C49B—Ru2B—P2B	100.33 (16)
C50A—Ru2A—P2A	91.35 (13)	C48B—Ru2B—P2B	92.98 (14)
C48A—Ru2A—P2A	89.58 (15)	C50B—Ru2B—P2B	90.74 (15)
C49A—Ru2A—Ru3A	96.33 (15)	C49B—Ru2B—Ru3B	100.09 (16)
C50A—Ru2A—Ru3A	95.05 (13)	C48B—Ru2B—Ru3B	94.22 (14)
C48A—Ru2A—Ru3A	81.88 (15)	C50B—Ru2B—Ru3B	80.34 (14)
P2A—Ru2A—Ru3A	160.28 (3)	P2B—Ru2B—Ru3B	157.84 (3)
C49A—Ru2A—Ru1A	154.29 (15)	C49B—Ru2B—Ru1B	156.86 (16)
C50A—Ru2A—Ru1A	77.77 (13)	C48B—Ru2B—Ru1B	77.34 (14)
C48A—Ru2A—Ru1A	95.24 (14)	C50B—Ru2B—Ru1B	96.49 (15)
P2A—Ru2A—Ru1A	102.82 (3)	P2B—Ru2B—Ru1B	101.39 (3)
Ru3A—Ru2A—Ru1A	60.664 (11)	Ru3B—Ru2B—Ru1B	60.057 (12)
C52A—Ru3A—C53A	92.0 (2)	C52B—Ru3B—C51B	93.8 (2)
C52A—Ru3A—C51A	91.2 (2)	C52B—Ru3B—C53B	90.71 (19)
C53A—Ru3A—C51A	176.3 (2)	C51B—Ru3B—C53B	174.62 (19)
C52A—Ru3A—Sb1A	103.07 (14)	C52B—Ru3B—Sb1B	101.26 (15)
C53A—Ru3A—Sb1A	90.78 (13)	C51B—Ru3B—Sb1B	90.55 (13)
C51A—Ru3A—Sb1A	90.36 (14)	C53B—Ru3B—Sb1B	91.52 (13)
C52A—Ru3A—Ru2A	95.94 (14)	C52B—Ru3B—Ru2B	98.10 (14)
C53A—Ru3A—Ru2A	96.45 (13)	C51B—Ru3B—Ru2B	94.91 (13)
C51A—Ru3A—Ru2A	81.31 (13)	C53B—Ru3B—Ru2B	81.48 (14)
Sb1A—Ru3A—Ru2A	159.415 (17)	Sb1B—Ru3B—Ru2B	159.491 (17)
C52A—Ru3A—Ru1A	154.47 (14)	C52B—Ru3B—Ru1B	156.09 (15)
C53A—Ru3A—Ru1A	81.75 (14)	C51B—Ru3B—Ru1B	78.56 (14)
C51A—Ru3A—Ru1A	94.55 (14)	C53B—Ru3B—Ru1B	96.16 (14)
Sb1A—Ru3A—Ru1A	101.740 (14)	Sb1B—Ru3B—Ru1B	101.418 (15)
Ru2A—Ru3A—Ru1A	60.576 (12)	Ru2B—Ru3B—Ru1B	60.604 (12)
C7A—P1A—C1A	103.1 (2)	C7B—P1B—C1B	102.2 (2)
C7A—P1A—C13A	103.4 (2)	C7B—P1B—C13B	103.6 (2)
C1A—P1A—C13A	100.7 (2)	C1B—P1B—C13B	100.9 (2)
C7A—P1A—Ru1A	115.41 (15)	C7B—P1B—Ru1B	115.21 (18)
C1A—P1A—Ru1A	116.31 (15)	C1B—P1B—Ru1B	116.72 (16)
C13A—P1A—Ru1A	115.84 (15)	C13B—P1B—Ru1B	116.02 (16)
C21A—P2A—C15A	105.1 (2)	C15B—P2B—C21B	102.8 (2)
C21A—P2A—C14A	102.0 (2)	C15B—P2B—C14B	103.2 (2)
C15A—P2A—C14A	102.9 (2)	C21B—P2B—C14B	100.9 (2)
C21A—P2A—Ru2A	114.15 (16)	C15B—P2B—Ru2B	114.64 (16)
C15A—P2A—Ru2A	115.98 (16)	C21B—P2B—Ru2B	118.02 (17)
C14A—P2A—Ru2A	115.03 (15)	C14B—P2B—Ru2B	115.19 (17)
C6A—C1A—C2A	118.7 (4)	C2B—C1B—C6B	117.6 (5)
C6A—C1A—P1A	122.8 (4)	C2B—C1B—P1B	123.1 (4)
C2A—C1A—P1A	118.5 (4)	C6B—C1B—P1B	119.2 (4)
C1A—C2A—C3A	120.6 (5)	C1B—C2B—C3B	122.0 (5)
C1A—C2A—H2AA	119.7	C1B—C2B—H2BA	119.0
C3A—C2A—H2AA	119.7	C3B—C2B—H2BA	119.0
C4A—C3A—C2A	119.8 (5)	C4B—C3B—C2B	119.6 (5)
C4A—C3A—H3AA	120.1	C4B—C3B—H3BA	120.2
C2A—C3A—H3AA	120.1	C2B—C3B—H3BA	120.2
C3A—C4A—C5A	120.4 (5)	C3B—C4B—C5B	120.0 (5)
C3A—C4A—H4AA	119.8	C3B—C4B—H4BA	120.0
C5A—C4A—H4AA	119.8	C5B—C4B—H4BA	120.0
C4A—C5A—C6A	119.6 (5)	C6B—C5B—C4B	120.1 (6)
C4A—C5A—H5AA	120.2	C6B—C5B—H5BA	120.0
C6A—C5A—H5AA	120.2	C4B—C5B—H5BA	120.0
C1A—C6A—C5A	121.0 (5)	C5B—C6B—C1B	120.6 (5)
C1A—C6A—H6AA	119.5	C5B—C6B—H6BA	119.7
C5A—C6A—H6AA	119.5	C1B—C6B—H6BA	119.7
C8A—C7A—C12A	117.1 (4)	C12B—C7B—C8B	118.3 (5)
C8A—C7A—P1A	119.9 (4)	C12B—C7B—P1B	123.4 (5)
C12A—C7A—P1A	123.0 (3)	C8B—C7B—P1B	118.3 (4)
C9A—C8A—C7A	122.0 (5)	C9B—C8B—C7B	121.2 (5)
C9A—C8A—H8AA	119.0	C9B—C8B—H8BA	119.4
C7A—C8A—H8AA	119.0	C7B—C8B—H8BA	119.4
C10A—C9A—C8A	120.1 (5)	C10B—C9B—C8B	119.7 (6)
C10A—C9A—H9AA	119.9	C10B—C9B—H9BA	120.1
C8A—C9A—H9AA	119.9	C8B—C9B—H9BA	120.1
C9A—C10A—C11A	119.3 (5)	C11B—C10B—C9B	120.3 (6)
C9A—C10A—H10A	120.3	C11B—C10B—H10B	119.9
C11A—C10A—H10A	120.3	C9B—C10B—H10B	119.9
C12A—C11A—C10A	120.8 (5)	C10B—C11B—C12B	120.6 (6)
C12A—C11A—H11A	119.6	C10B—C11B—H11B	119.7
C10A—C11A—H11A	119.6	C12B—C11B—H11B	119.7
C11A—C12A—C7A	120.6 (5)	C7B—C12B—C11B	119.9 (6)
C11A—C12A—H12A	119.7	C7B—C12B—H12B	120.0
C7A—C12A—H12A	119.7	C11B—C12B—H12B	120.0
C14A—C13A—P1A	113.1 (3)	C14B—C13B—P1B	113.1 (3)
C14A—C13A—H13A	109.0	C14B—C13B—H13C	109.0
P1A—C13A—H13A	109.0	P1B—C13B—H13C	109.0
C14A—C13A—H13B	109.0	C14B—C13B—H13D	109.0
P1A—C13A—H13B	109.0	P1B—C13B—H13D	109.0
H13A—C13A—H13B	107.8	H13C—C13B—H13D	107.8
C13A—C14A—P2A	112.5 (3)	C13B—C14B—P2B	112.8 (4)
C13A—C14A—H14A	109.1	C13B—C14B—H14C	109.0
P2A—C14A—H14A	109.1	P2B—C14B—H14C	109.0
C13A—C14A—H14B	109.1	C13B—C14B—H14D	109.0
P2A—C14A—H14B	109.1	P2B—C14B—H14D	109.0
H14A—C14A—H14B	107.8	H14C—C14B—H14D	107.8
C20A—C15A—C16A	118.0 (4)	C20B—C15B—C16B	118.1 (5)
C20A—C15A—P2A	121.3 (4)	C20B—C15B—P2B	122.2 (4)
C16A—C15A—P2A	120.7 (4)	C16B—C15B—P2B	119.5 (4)
C17A—C16A—C15A	121.3 (5)	C17B—C16B—C15B	121.4 (5)
C17A—C16A—H16A	119.4	C17B—C16B—H16B	119.3
C15A—C16A—H16A	119.4	C15B—C16B—H16B	119.3
C18A—C17A—C16A	120.2 (6)	C18B—C17B—C16B	119.5 (6)
C18A—C17A—H17A	119.9	C18B—C17B—H17B	120.3
C16A—C17A—H17A	119.9	C16B—C17B—H17B	120.3
C17A—C18A—C19A	120.1 (5)	C19B—C18B—C17B	119.9 (6)
C17A—C18A—H18A	120.0	C19B—C18B—H18B	120.1
C19A—C18A—H18A	120.0	C17B—C18B—H18B	120.1
C18A—C19A—C20A	119.9 (5)	C18B—C19B—C20B	120.4 (6)
C18A—C19A—H19A	120.0	C18B—C19B—H19B	119.8
C20A—C19A—H19A	120.0	C20B—C19B—H19B	119.8
C15A—C20A—C19A	120.5 (5)	C19B—C20B—C15B	120.8 (6)
C15A—C20A—H20A	119.7	C19B—C20B—H20B	119.6
C19A—C20A—H20A	119.7	C15B—C20B—H20B	119.6
C26A—C21A—C22A	116.7 (5)	C22B—C21B—C26B	119.1 (5)
C26A—C21A—P2A	121.2 (4)	C22B—C21B—P2B	121.8 (4)
C22A—C21A—P2A	121.9 (4)	C26B—C21B—P2B	119.1 (4)
C23A—C22A—C21A	121.1 (6)	C21B—C22B—C23B	120.3 (6)
C23A—C22A—H22A	119.4	C21B—C22B—H22B	119.9
C21A—C22A—H22A	119.4	C23B—C22B—H22B	119.9
C24A—C23A—C22A	121.5 (6)	C24B—C23B—C22B	120.7 (6)
C24A—C23A—H23A	119.3	C24B—C23B—H23B	119.6
C22A—C23A—H23A	119.3	C22B—C23B—H23B	119.6
C23A—C24A—C25A	118.2 (6)	C23B—C24B—C25B	119.8 (5)
C23A—C24A—H24A	120.9	C23B—C24B—H24B	120.1
C25A—C24A—H24A	120.9	C25B—C24B—H24B	120.1
C26A—C25A—C24A	120.9 (6)	C26B—C25B—C24B	119.7 (6)
C26A—C25A—H25A	119.5	C26B—C25B—H25B	120.1
C24A—C25A—H25A	119.5	C24B—C25B—H25B	120.1
C25A—C26A—C21A	121.5 (6)	C25B—C26B—C21B	120.4 (6)
C25A—C26A—H26A	119.2	C25B—C26B—H26B	119.8
C21A—C26A—H26A	119.2	C21B—C26B—H26B	119.8
C28A—C27A—C32A	118.7 (5)	C28B—C27B—C32B	118.5 (5)
C28A—C27A—Sb1A	115.7 (4)	C28B—C27B—Sb1B	121.6 (4)
C32A—C27A—Sb1A	125.2 (4)	C32B—C27B—Sb1B	119.9 (3)
C27A—C28A—C29A	121.8 (5)	C27B—C28B—C29B	120.4 (5)
C27A—C28A—H28A	119.1	C27B—C28B—H28B	119.8
C29A—C28A—H28A	119.1	C29B—C28B—H28B	119.8
C30A—C29A—C28A	119.3 (5)	C30B—C29B—C28B	121.1 (5)
C30A—C29A—H29A	120.3	C30B—C29B—H29B	119.5
C28A—C29A—H29A	120.3	C28B—C29B—H29B	119.5
C29A—C30A—C31A	119.5 (5)	C29B—C30B—C31B	119.1 (5)
C29A—C30A—H30A	120.3	C29B—C30B—H30B	120.5
C31A—C30A—H30A	120.3	C31B—C30B—H30B	120.5
C32A—C31A—C30A	120.8 (5)	C32B—C31B—C30B	120.2 (5)
C32A—C31A—H31A	119.6	C32B—C31B—H31B	119.9
C30A—C31A—H31A	119.6	C30B—C31B—H31B	119.9
C27A—C32A—C31A	119.9 (5)	C31B—C32B—C27B	120.6 (5)
C27A—C32A—H32A	120.0	C31B—C32B—H32B	119.7
C31A—C32A—H32A	120.0	C27B—C32B—H32B	119.7
C38A—C33A—C34A	118.1 (5)	C34B—C33B—C38B	119.4 (4)
C38A—C33A—Sb1A	118.1 (4)	C34B—C33B—Sb1B	119.4 (3)
C34A—C33A—Sb1A	123.4 (4)	C38B—C33B—Sb1B	120.5 (3)
C35A—C34A—C33A	120.3 (5)	C33B—C34B—C35B	121.0 (4)
C35A—C34A—H34A	119.8	C33B—C34B—H34B	119.5
C33A—C34A—H34A	119.8	C35B—C34B—H34B	119.5
C34A—C35A—C36A	119.9 (5)	C36B—C35B—C34B	119.4 (5)
C34A—C35A—H35A	120.0	C36B—C35B—H35B	120.3
C36A—C35A—H35A	120.0	C34B—C35B—H35B	120.3
C37A—C36A—C35A	119.4 (5)	C37B—C36B—C35B	120.1 (4)
C37A—C36A—H36A	120.3	C37B—C36B—H36B	120.0
C35A—C36A—H36A	120.3	C35B—C36B—H36B	120.0
C36A—C37A—C38A	120.8 (5)	C36B—C37B—C38B	120.5 (5)
C36A—C37A—H37A	119.6	C36B—C37B—H37B	119.8
C38A—C37A—H37A	119.6	C38B—C37B—H37B	119.8
C33A—C38A—C37A	121.2 (5)	C37B—C38B—C33B	119.5 (5)
C33A—C38A—H38A	119.4	C37B—C38B—H38B	120.2
C37A—C38A—H38A	119.4	C33B—C38B—H38B	120.2
C40A—C39A—C44A	120.0 (5)	C40B—C39B—C44B	119.0 (4)
C40A—C39A—Sb1A	120.3 (3)	C40B—C39B—Sb1B	123.0 (3)
C44A—C39A—Sb1A	119.7 (4)	C44B—C39B—Sb1B	117.6 (3)
C39A—C40A—C41A	120.2 (5)	C39B—C40B—C41B	120.5 (5)
C39A—C40A—H40A	119.9	C39B—C40B—H40B	119.7
C41A—C40A—H40A	119.9	C41B—C40B—H40B	119.7
C42A—C41A—C40A	119.8 (5)	C42B—C41B—C40B	119.9 (5)
C42A—C41A—H41A	120.1	C42B—C41B—H41B	120.1
C40A—C41A—H41A	120.1	C40B—C41B—H41B	120.1
C43A—C42A—C41A	120.1 (5)	C41B—C42B—C43B	120.4 (5)
C43A—C42A—H42A	120.0	C41B—C42B—H42B	119.8
C41A—C42A—H42A	120.0	C43B—C42B—H42B	119.8
C42A—C43A—C44A	120.2 (5)	C42B—C43B—C44B	120.0 (5)
C42A—C43A—H43A	119.9	C42B—C43B—H43B	120.0
C44A—C43A—H43A	119.9	C44B—C43B—H43B	120.0
C43A—C44A—C39A	119.8 (5)	C43B—C44B—C39B	120.2 (5)
C43A—C44A—H44A	120.1	C43B—C44B—H44B	119.9
C39A—C44A—H44A	120.1	C39B—C44B—H44B	119.9
O3A—C45A—Ru1A	172.0 (4)	O1B—C45B—Ru1B	172.1 (4)
O2A—C46A—Ru1A	175.3 (4)	O2B—C46B—Ru1B	175.8 (4)
O1A—C47A—Ru1A	174.6 (4)	O3B—C47B—Ru1B	173.8 (4)
O4A—C48A—Ru2A	174.6 (4)	O4B—C48B—Ru2B	173.6 (4)
O5A—C49A—Ru2A	177.1 (5)	O5B—C49B—Ru2B	177.5 (6)
O6A—C50A—Ru2A	172.8 (4)	O6B—C50B—Ru2B	175.6 (5)
O7A—C51A—Ru3A	174.5 (4)	O7B—C51B—Ru3B	175.8 (4)
O8A—C52A—Ru3A	176.7 (4)	O8B—C52B—Ru3B	178.0 (4)
O9A—C53A—Ru3A	174.8 (4)	O9B—C53B—Ru3B	174.4 (4)
			
C46A—Ru1A—Ru2A—C49A	60.7 (5)	C46B—Ru1B—Ru2B—C49B	−71.4 (6)
C45A—Ru1A—Ru2A—C49A	130.7 (4)	C45B—Ru1B—Ru2B—C49B	−137.1 (5)
C47A—Ru1A—Ru2A—C49A	−48.2 (4)	C47B—Ru1B—Ru2B—C49B	39.4 (5)
P1A—Ru1A—Ru2A—C49A	−142.9 (4)	P1B—Ru1B—Ru2B—C49B	132.0 (5)
Ru3A—Ru1A—Ru2A—C49A	28.9 (4)	Ru3B—Ru1B—Ru2B—C49B	−33.5 (5)
C46A—Ru1A—Ru2A—C50A	134.8 (4)	C46B—Ru1B—Ru2B—C48B	−140.4 (4)
C45A—Ru1A—Ru2A—C50A	−155.1 (2)	C45B—Ru1B—Ru2B—C48B	153.9 (2)
C47A—Ru1A—Ru2A—C50A	25.95 (19)	C47B—Ru1B—Ru2B—C48B	−29.6 (2)
P1A—Ru1A—Ru2A—C50A	−68.71 (14)	P1B—Ru1B—Ru2B—C48B	63.06 (15)
Ru3A—Ru1A—Ru2A—C50A	103.01 (13)	Ru3B—Ru1B—Ru2B—C48B	−102.50 (15)
C46A—Ru1A—Ru2A—C48A	−45.8 (4)	C46B—Ru1B—Ru2B—C50B	37.0 (4)
C45A—Ru1A—Ru2A—C48A	24.2 (2)	C45B—Ru1B—Ru2B—C50B	−28.7 (2)
C47A—Ru1A—Ru2A—C48A	−154.7 (2)	C47B—Ru1B—Ru2B—C50B	147.8 (2)
P1A—Ru1A—Ru2A—C48A	110.64 (16)	P1B—Ru1B—Ru2B—C50B	−119.54 (16)
Ru3A—Ru1A—Ru2A—C48A	−77.63 (16)	Ru3B—Ru1B—Ru2B—C50B	74.90 (16)
C46A—Ru1A—Ru2A—P2A	−136.6 (3)	C46B—Ru1B—Ru2B—P2B	129.1 (4)
C45A—Ru1A—Ru2A—P2A	−66.54 (15)	C45B—Ru1B—Ru2B—P2B	63.38 (13)
C47A—Ru1A—Ru2A—P2A	114.54 (14)	C47B—Ru1B—Ru2B—P2B	−120.11 (15)
P1A—Ru1A—Ru2A—P2A	19.88 (4)	P1B—Ru1B—Ru2B—P2B	−27.47 (5)
Ru3A—Ru1A—Ru2A—P2A	−168.40 (3)	Ru3B—Ru1B—Ru2B—P2B	166.97 (3)
C46A—Ru1A—Ru2A—Ru3A	31.8 (3)	C46B—Ru1B—Ru2B—Ru3B	−37.9 (4)
C45A—Ru1A—Ru2A—Ru3A	101.86 (15)	C45B—Ru1B—Ru2B—Ru3B	−103.59 (13)
C47A—Ru1A—Ru2A—Ru3A	−77.06 (14)	C47B—Ru1B—Ru2B—Ru3B	72.91 (14)
P1A—Ru1A—Ru2A—Ru3A	−171.72 (3)	P1B—Ru1B—Ru2B—Ru3B	165.56 (4)
C33A—Sb1A—Ru3A—C52A	88.6 (2)	C39B—Sb1B—Ru3B—C52B	140.72 (19)
C39A—Sb1A—Ru3A—C52A	−28.4 (2)	C27B—Sb1B—Ru3B—C52B	27.51 (19)
C27A—Sb1A—Ru3A—C52A	−141.3 (2)	C33B—Sb1B—Ru3B—C52B	−92.4 (2)
C33A—Sb1A—Ru3A—C53A	−3.6 (2)	C39B—Sb1B—Ru3B—C51B	−125.34 (19)
C39A—Sb1A—Ru3A—C53A	−120.60 (19)	C27B—Sb1B—Ru3B—C51B	121.45 (19)
C27A—Sb1A—Ru3A—C53A	126.5 (2)	C33B—Sb1B—Ru3B—C51B	1.6 (2)
C33A—Sb1A—Ru3A—C51A	179.9 (2)	C39B—Sb1B—Ru3B—C53B	49.70 (19)
C39A—Sb1A—Ru3A—C51A	62.94 (19)	C27B—Sb1B—Ru3B—C53B	−63.52 (19)
C27A—Sb1A—Ru3A—C51A	−50.0 (2)	C33B—Sb1B—Ru3B—C53B	176.6 (2)
C33A—Sb1A—Ru3A—Ru2A	−114.47 (16)	C39B—Sb1B—Ru3B—Ru2B	−19.69 (14)
C39A—Sb1A—Ru3A—Ru2A	128.56 (14)	C27B—Sb1B—Ru3B—Ru2B	−132.91 (14)
C27A—Sb1A—Ru3A—Ru2A	15.66 (15)	C33B—Sb1B—Ru3B—Ru2B	107.22 (15)
C33A—Sb1A—Ru3A—Ru1A	−85.37 (16)	C39B—Sb1B—Ru3B—Ru1B	−46.90 (13)
C39A—Sb1A—Ru3A—Ru1A	157.66 (14)	C27B—Sb1B—Ru3B—Ru1B	−160.11 (13)
C27A—Sb1A—Ru3A—Ru1A	44.77 (14)	C33B—Sb1B—Ru3B—Ru1B	80.02 (15)
C49A—Ru2A—Ru3A—C52A	22.8 (2)	C49B—Ru2B—Ru3B—C52B	−24.3 (2)
C50A—Ru2A—Ru3A—C52A	117.7 (2)	C48B—Ru2B—Ru3B—C52B	−118.8 (2)
C48A—Ru2A—Ru3A—C52A	−68.7 (2)	C50B—Ru2B—Ru3B—C52B	65.1 (2)
P2A—Ru2A—Ru3A—C52A	−133.87 (18)	P2B—Ru2B—Ru3B—C52B	132.58 (17)
Ru1A—Ru2A—Ru3A—C52A	−169.41 (15)	Ru1B—Ru2B—Ru3B—C52B	168.44 (14)
C49A—Ru2A—Ru3A—C53A	115.4 (2)	C49B—Ru2B—Ru3B—C51B	−118.8 (2)
C50A—Ru2A—Ru3A—C53A	−149.66 (19)	C48B—Ru2B—Ru3B—C51B	146.7 (2)
C48A—Ru2A—Ru3A—C53A	24.0 (2)	C50B—Ru2B—Ru3B—C51B	−29.4 (2)
P2A—Ru2A—Ru3A—C53A	−41.19 (17)	P2B—Ru2B—Ru3B—C51B	38.07 (17)
Ru1A—Ru2A—Ru3A—C53A	−76.73 (14)	Ru1B—Ru2B—Ru3B—C51B	73.93 (14)
C49A—Ru2A—Ru3A—C51A	−67.6 (2)	C49B—Ru2B—Ru3B—C53B	65.2 (2)
C50A—Ru2A—Ru3A—C51A	27.35 (19)	C48B—Ru2B—Ru3B—C53B	−29.3 (2)
C48A—Ru2A—Ru3A—C51A	−159.0 (2)	C50B—Ru2B—Ru3B—C53B	154.6 (2)
P2A—Ru2A—Ru3A—C51A	135.82 (17)	P2B—Ru2B—Ru3B—C53B	−137.92 (16)
Ru1A—Ru2A—Ru3A—C51A	100.27 (14)	Ru1B—Ru2B—Ru3B—C53B	−102.06 (14)
C49A—Ru2A—Ru3A—Sb1A	−134.69 (17)	C49B—Ru2B—Ru3B—Sb1B	136.30 (19)
C50A—Ru2A—Ru3A—Sb1A	−39.78 (14)	C48B—Ru2B—Ru3B—Sb1B	41.82 (15)
C48A—Ru2A—Ru3A—Sb1A	133.86 (16)	C50B—Ru2B—Ru3B—Sb1B	−134.28 (17)
P2A—Ru2A—Ru3A—Sb1A	68.69 (11)	P2B—Ru2B—Ru3B—Sb1B	−66.82 (11)
Ru1A—Ru2A—Ru3A—Sb1A	33.14 (5)	Ru1B—Ru2B—Ru3B—Sb1B	−30.96 (5)
C49A—Ru2A—Ru3A—Ru1A	−167.83 (16)	C49B—Ru2B—Ru3B—Ru1B	167.26 (19)
C50A—Ru2A—Ru3A—Ru1A	−72.93 (13)	C48B—Ru2B—Ru3B—Ru1B	72.77 (14)
C48A—Ru2A—Ru3A—Ru1A	100.72 (15)	C50B—Ru2B—Ru3B—Ru1B	−103.32 (16)
P2A—Ru2A—Ru3A—Ru1A	35.55 (9)	P2B—Ru2B—Ru3B—Ru1B	−35.86 (9)
C46A—Ru1A—Ru3A—C52A	−141.6 (4)	C46B—Ru1B—Ru3B—C52B	136.6 (4)
C45A—Ru1A—Ru3A—C52A	−48.9 (4)	C45B—Ru1B—Ru3B—C52B	42.3 (3)
C47A—Ru1A—Ru3A—C52A	127.2 (4)	C47B—Ru1B—Ru3B—C52B	−132.2 (4)
P1A—Ru1A—Ru3A—C52A	48.2 (3)	P1B—Ru1B—Ru3B—C52B	−67.2 (3)
Ru2A—Ru1A—Ru3A—C52A	25.1 (3)	Ru2B—Ru1B—Ru3B—C52B	−29.3 (3)
C46A—Ru1A—Ru3A—C53A	−64.46 (19)	C46B—Ru1B—Ru3B—C51B	63.6 (2)
C45A—Ru1A—Ru3A—C53A	28.29 (19)	C45B—Ru1B—Ru3B—C51B	−30.74 (18)
C47A—Ru1A—Ru3A—C53A	−155.60 (19)	C47B—Ru1B—Ru3B—C51B	154.71 (19)
P1A—Ru1A—Ru3A—C53A	125.32 (16)	P1B—Ru1B—Ru3B—C51B	−140.27 (16)
Ru2A—Ru1A—Ru3A—C53A	102.24 (14)	Ru2B—Ru1B—Ru3B—C51B	−102.37 (13)
C46A—Ru1A—Ru3A—C51A	115.9 (2)	C46B—Ru1B—Ru3B—C53B	−117.4 (2)
C45A—Ru1A—Ru3A—C51A	−151.31 (19)	C45B—Ru1B—Ru3B—C53B	148.22 (18)
C47A—Ru1A—Ru3A—C51A	24.80 (19)	C47B—Ru1B—Ru3B—C53B	−26.33 (19)
P1A—Ru1A—Ru3A—C51A	−54.28 (17)	P1B—Ru1B—Ru3B—C53B	38.69 (17)
Ru2A—Ru1A—Ru3A—C51A	−77.36 (14)	Ru2B—Ru1B—Ru3B—C53B	76.60 (13)
C46A—Ru1A—Ru3A—Sb1A	24.62 (14)	C46B—Ru1B—Ru3B—Sb1B	−24.63 (14)
C45A—Ru1A—Ru3A—Sb1A	117.37 (13)	C45B—Ru1B—Ru3B—Sb1B	−118.97 (13)
C47A—Ru1A—Ru3A—Sb1A	−66.52 (13)	C47B—Ru1B—Ru3B—Sb1B	66.48 (14)
P1A—Ru1A—Ru3A—Sb1A	−145.60 (9)	P1B—Ru1B—Ru3B—Sb1B	131.50 (9)
Ru2A—Ru1A—Ru3A—Sb1A	−168.678 (17)	Ru2B—Ru1B—Ru3B—Sb1B	169.406 (17)
C46A—Ru1A—Ru3A—Ru2A	−166.70 (14)	C46B—Ru1B—Ru3B—Ru2B	165.96 (14)
C45A—Ru1A—Ru3A—Ru2A	−73.95 (13)	C45B—Ru1B—Ru3B—Ru2B	71.62 (13)
C47A—Ru1A—Ru3A—Ru2A	102.15 (13)	C47B—Ru1B—Ru3B—Ru2B	−102.93 (14)
P1A—Ru1A—Ru3A—Ru2A	23.07 (9)	P1B—Ru1B—Ru3B—Ru2B	−37.91 (9)
C46A—Ru1A—P1A—C7A	48.9 (2)	C46B—Ru1B—P1B—C7B	74.8 (2)
C45A—Ru1A—P1A—C7A	−43.0 (2)	C45B—Ru1B—P1B—C7B	168.7 (2)
C47A—Ru1A—P1A—C7A	141.9 (2)	C47B—Ru1B—P1B—C7B	−19.1 (2)
Ru2A—Ru1A—P1A—C7A	−121.00 (17)	Ru3B—Ru1B—P1B—C7B	−81.6 (2)
Ru3A—Ru1A—P1A—C7A	−141.00 (17)	Ru2B—Ru1B—P1B—C7B	−114.18 (17)
C46A—Ru1A—P1A—C1A	−72.1 (2)	C46B—Ru1B—P1B—C1B	−45.1 (2)
C45A—Ru1A—P1A—C1A	−164.1 (2)	C45B—Ru1B—P1B—C1B	48.8 (2)
C47A—Ru1A—P1A—C1A	20.9 (2)	C47B—Ru1B—P1B—C1B	−139.0 (2)
Ru2A—Ru1A—P1A—C1A	117.99 (16)	Ru3B—Ru1B—P1B—C1B	158.53 (19)
Ru3A—Ru1A—P1A—C1A	97.98 (18)	Ru2B—Ru1B—P1B—C1B	125.9 (2)
C46A—Ru1A—P1A—C13A	169.8 (2)	C46B—Ru1B—P1B—C13B	−163.9 (2)
C45A—Ru1A—P1A—C13A	77.9 (2)	C45B—Ru1B—P1B—C13B	−70.0 (2)
C47A—Ru1A—P1A—C13A	−97.1 (2)	C47B—Ru1B—P1B—C13B	102.2 (2)
Ru2A—Ru1A—P1A—C13A	−0.03 (17)	Ru3B—Ru1B—P1B—C13B	39.8 (2)
Ru3A—Ru1A—P1A—C13A	−20.0 (2)	Ru2B—Ru1B—P1B—C13B	7.1 (2)
C49A—Ru2A—P2A—C21A	−68.3 (2)	C49B—Ru2B—P2B—C15B	72.9 (3)
C50A—Ru2A—P2A—C21A	−163.0 (2)	C48B—Ru2B—P2B—C15B	167.2 (2)
C48A—Ru2A—P2A—C21A	24.0 (2)	C50B—Ru2B—P2B—C15B	−18.4 (2)
Ru3A—Ru2A—P2A—C21A	87.96 (19)	Ru3B—Ru2B—P2B—C15B	−84.0 (2)
Ru1A—Ru2A—P2A—C21A	119.28 (17)	Ru1B—Ru2B—P2B—C15B	−115.14 (18)
C49A—Ru2A—P2A—C15A	54.1 (2)	C49B—Ru2B—P2B—C21B	−48.4 (3)
C50A—Ru2A—P2A—C15A	−40.5 (2)	C48B—Ru2B—P2B—C21B	45.8 (2)
C48A—Ru2A—P2A—C15A	146.4 (2)	C50B—Ru2B—P2B—C21B	−139.7 (2)
Ru3A—Ru2A—P2A—C15A	−149.62 (16)	Ru3B—Ru2B—P2B—C21B	154.70 (17)
Ru1A—Ru2A—P2A—C15A	−118.30 (16)	Ru1B—Ru2B—P2B—C21B	123.51 (18)
C49A—Ru2A—P2A—C14A	174.2 (2)	C49B—Ru2B—P2B—C14B	−167.6 (3)
C50A—Ru2A—P2A—C14A	79.6 (2)	C48B—Ru2B—P2B—C14B	−73.3 (2)
C48A—Ru2A—P2A—C14A	−93.5 (2)	C50B—Ru2B—P2B—C14B	101.1 (2)
Ru3A—Ru2A—P2A—C14A	−29.5 (2)	Ru3B—Ru2B—P2B—C14B	35.6 (2)
Ru1A—Ru2A—P2A—C14A	1.80 (16)	Ru1B—Ru2B—P2B—C14B	4.38 (19)
C7A—P1A—C1A—C6A	−20.2 (4)	C7B—P1B—C1B—C2B	−125.4 (5)
C13A—P1A—C1A—C6A	−126.8 (4)	C13B—P1B—C1B—C2B	127.9 (5)
Ru1A—P1A—C1A—C6A	107.2 (4)	Ru1B—P1B—C1B—C2B	1.3 (5)
C7A—P1A—C1A—C2A	161.4 (4)	C7B—P1B—C1B—C6B	50.9 (5)
C13A—P1A—C1A—C2A	54.8 (4)	C13B—P1B—C1B—C6B	−55.8 (5)
Ru1A—P1A—C1A—C2A	−71.2 (4)	Ru1B—P1B—C1B—C6B	177.5 (4)
C6A—C1A—C2A—C3A	0.7 (7)	C6B—C1B—C2B—C3B	1.6 (8)
P1A—C1A—C2A—C3A	179.1 (4)	P1B—C1B—C2B—C3B	177.9 (4)
C1A—C2A—C3A—C4A	0.1 (9)	C1B—C2B—C3B—C4B	0.6 (9)
C2A—C3A—C4A—C5A	−1.1 (9)	C2B—C3B—C4B—C5B	−2.0 (10)
C3A—C4A—C5A—C6A	1.2 (9)	C3B—C4B—C5B—C6B	1.1 (10)
C2A—C1A—C6A—C5A	−0.5 (7)	C4B—C5B—C6B—C1B	1.2 (10)
P1A—C1A—C6A—C5A	−178.9 (4)	C2B—C1B—C6B—C5B	−2.5 (9)
C4A—C5A—C6A—C1A	−0.4 (8)	P1B—C1B—C6B—C5B	−178.9 (5)
C1A—P1A—C7A—C8A	84.7 (4)	C1B—P1B—C7B—C12B	−92.3 (5)
C13A—P1A—C7A—C8A	−170.8 (4)	C13B—P1B—C7B—C12B	12.2 (5)
Ru1A—P1A—C7A—C8A	−43.3 (5)	Ru1B—P1B—C7B—C12B	140.1 (4)
C1A—P1A—C7A—C12A	−94.4 (4)	C1B—P1B—C7B—C8B	86.8 (4)
C13A—P1A—C7A—C12A	10.2 (5)	C13B—P1B—C7B—C8B	−168.6 (4)
Ru1A—P1A—C7A—C12A	137.7 (4)	Ru1B—P1B—C7B—C8B	−40.8 (4)
C12A—C7A—C8A—C9A	1.8 (8)	C12B—C7B—C8B—C9B	−0.2 (8)
P1A—C7A—C8A—C9A	−177.3 (5)	P1B—C7B—C8B—C9B	−179.4 (4)
C7A—C8A—C9A—C10A	−1.2 (9)	C7B—C8B—C9B—C10B	0.5 (9)
C8A—C9A—C10A—C11A	−0.5 (9)	C8B—C9B—C10B—C11B	0.5 (11)
C9A—C10A—C11A—C12A	1.4 (9)	C9B—C10B—C11B—C12B	−1.9 (11)
C10A—C11A—C12A—C7A	−0.8 (9)	C8B—C7B—C12B—C11B	−1.2 (9)
C8A—C7A—C12A—C11A	−0.8 (8)	P1B—C7B—C12B—C11B	178.0 (5)
P1A—C7A—C12A—C11A	178.2 (4)	C10B—C11B—C12B—C7B	2.2 (11)
C7A—P1A—C13A—C14A	69.4 (3)	C7B—P1B—C13B—C14B	−179.1 (4)
C1A—P1A—C13A—C14A	175.8 (3)	C1B—P1B—C13B—C14B	−73.6 (4)
Ru1A—P1A—C13A—C14A	−57.8 (3)	Ru1B—P1B—C13B—C14B	53.5 (4)
P1A—C13A—C14A—P2A	98.9 (3)	P1B—C13B—C14B—P2B	−97.8 (4)
C21A—P2A—C14A—C13A	175.7 (3)	C15B—P2B—C14B—C13B	−177.1 (3)
C15A—P2A—C14A—C13A	66.9 (3)	C21B—P2B—C14B—C13B	−71.0 (4)
Ru2A—P2A—C14A—C13A	−60.2 (3)	Ru2B—P2B—C14B—C13B	57.2 (4)
C21A—P2A—C15A—C20A	−75.2 (5)	C21B—P2B—C15B—C20B	−148.0 (4)
C14A—P2A—C15A—C20A	31.2 (5)	C14B—P2B—C15B—C20B	−43.4 (5)
Ru2A—P2A—C15A—C20A	157.7 (4)	Ru2B—P2B—C15B—C20B	82.6 (5)
C21A—P2A—C15A—C16A	106.1 (4)	C21B—P2B—C15B—C16B	37.0 (5)
C14A—P2A—C15A—C16A	−147.5 (4)	C14B—P2B—C15B—C16B	141.6 (4)
Ru2A—P2A—C15A—C16A	−21.0 (4)	Ru2B—P2B—C15B—C16B	−92.3 (4)
C20A—C15A—C16A—C17A	0.5 (7)	C20B—C15B—C16B—C17B	0.0 (8)
P2A—C15A—C16A—C17A	179.3 (4)	P2B—C15B—C16B—C17B	175.2 (4)
C15A—C16A—C17A—C18A	−1.8 (8)	C15B—C16B—C17B—C18B	0.2 (9)
C16A—C17A—C18A—C19A	1.6 (8)	C16B—C17B—C18B—C19B	−0.7 (9)
C17A—C18A—C19A—C20A	−0.1 (9)	C17B—C18B—C19B—C20B	1.1 (9)
C16A—C15A—C20A—C19A	1.0 (8)	C18B—C19B—C20B—C15B	−0.9 (9)
P2A—C15A—C20A—C19A	−177.7 (4)	C16B—C15B—C20B—C19B	0.3 (8)
C18A—C19A—C20A—C15A	−1.2 (9)	P2B—C15B—C20B—C19B	−174.7 (4)
C15A—P2A—C21A—C26A	−38.5 (5)	C15B—P2B—C21B—C22B	−125.0 (5)
C14A—P2A—C21A—C26A	−145.6 (5)	C14B—P2B—C21B—C22B	128.6 (5)
Ru2A—P2A—C21A—C26A	89.7 (5)	Ru2B—P2B—C21B—C22B	2.2 (5)
C15A—P2A—C21A—C22A	147.9 (5)	C15B—P2B—C21B—C26B	55.0 (5)
C14A—P2A—C21A—C22A	40.8 (6)	C14B—P2B—C21B—C26B	−51.3 (5)
Ru2A—P2A—C21A—C22A	−83.9 (6)	Ru2B—P2B—C21B—C26B	−177.7 (4)
C26A—C21A—C22A—C23A	−1.6 (11)	C26B—C21B—C22B—C23B	1.2 (9)
P2A—C21A—C22A—C23A	172.3 (7)	P2B—C21B—C22B—C23B	−178.7 (5)
C21A—C22A—C23A—C24A	1.9 (14)	C21B—C22B—C23B—C24B	0.2 (11)
C22A—C23A—C24A—C25A	−1.4 (13)	C22B—C23B—C24B—C25B	−1.5 (11)
C23A—C24A—C25A—C26A	0.6 (12)	C23B—C24B—C25B—C26B	1.4 (11)
C24A—C25A—C26A—C21A	−0.3 (12)	C24B—C25B—C26B—C21B	0.0 (10)
C22A—C21A—C26A—C25A	0.8 (10)	C22B—C21B—C26B—C25B	−1.3 (9)
P2A—C21A—C26A—C25A	−173.1 (6)	P2B—C21B—C26B—C25B	178.6 (5)
C33A—Sb1A—C27A—C28A	−143.9 (4)	C39B—Sb1B—C27B—C28B	78.7 (5)
C39A—Sb1A—C27A—C28A	−43.4 (4)	C33B—Sb1B—C27B—C28B	−24.9 (5)
Ru3A—Sb1A—C27A—C28A	79.3 (4)	Ru3B—Sb1B—C27B—C28B	−158.7 (4)
C33A—Sb1A—C27A—C32A	43.4 (5)	C39B—Sb1B—C27B—C32B	−100.1 (4)
C39A—Sb1A—C27A—C32A	143.9 (4)	C33B—Sb1B—C27B—C32B	156.3 (4)
Ru3A—Sb1A—C27A—C32A	−93.3 (4)	Ru3B—Sb1B—C27B—C32B	22.5 (4)
C32A—C27A—C28A—C29A	0.6 (8)	C32B—C27B—C28B—C29B	2.1 (9)
Sb1A—C27A—C28A—C29A	−172.5 (4)	Sb1B—C27B—C28B—C29B	−176.8 (5)
C27A—C28A—C29A—C30A	−0.4 (8)	C27B—C28B—C29B—C30B	−1.1 (11)
C28A—C29A—C30A—C31A	0.3 (9)	C28B—C29B—C30B—C31B	−0.1 (10)
C29A—C30A—C31A—C32A	−0.4 (10)	C29B—C30B—C31B—C32B	0.3 (9)
C28A—C27A—C32A—C31A	−0.7 (8)	C30B—C31B—C32B—C27B	0.7 (9)
Sb1A—C27A—C32A—C31A	171.8 (4)	C28B—C27B—C32B—C31B	−1.9 (8)
C30A—C31A—C32A—C27A	0.6 (9)	Sb1B—C27B—C32B—C31B	177.0 (4)
C39A—Sb1A—C33A—C38A	39.5 (5)	C39B—Sb1B—C33B—C34B	170.1 (4)
C27A—Sb1A—C33A—C38A	139.3 (5)	C27B—Sb1B—C33B—C34B	−89.4 (4)
Ru3A—Sb1A—C33A—C38A	−85.9 (5)	Ru3B—Sb1B—C33B—C34B	37.9 (4)
C39A—Sb1A—C33A—C34A	−147.6 (4)	C39B—Sb1B—C33B—C38B	−19.3 (4)
C27A—Sb1A—C33A—C34A	−47.7 (5)	C27B—Sb1B—C33B—C38B	81.2 (4)
Ru3A—Sb1A—C33A—C34A	87.0 (4)	Ru3B—Sb1B—C33B—C38B	−151.5 (3)
C38A—C33A—C34A—C35A	−4.1 (9)	C38B—C33B—C34B—C35B	0.4 (7)
Sb1A—C33A—C34A—C35A	−177.0 (4)	Sb1B—C33B—C34B—C35B	171.1 (4)
C33A—C34A—C35A—C36A	1.2 (9)	C33B—C34B—C35B—C36B	−1.0 (8)
C34A—C35A—C36A—C37A	3.6 (10)	C34B—C35B—C36B—C37B	1.1 (8)
C35A—C36A—C37A—C38A	−5.3 (11)	C35B—C36B—C37B—C38B	−0.7 (8)
C34A—C33A—C38A—C37A	2.4 (10)	C36B—C37B—C38B—C33B	0.1 (8)
Sb1A—C33A—C38A—C37A	175.7 (5)	C34B—C33B—C38B—C37B	0.0 (8)
C36A—C37A—C38A—C33A	2.4 (11)	Sb1B—C33B—C38B—C37B	−170.6 (4)
C33A—Sb1A—C39A—C40A	−122.7 (4)	C27B—Sb1B—C39B—C40B	−146.7 (4)
C27A—Sb1A—C39A—C40A	130.3 (4)	C33B—Sb1B—C39B—C40B	−46.7 (4)
Ru3A—Sb1A—C39A—C40A	6.4 (4)	Ru3B—Sb1B—C39B—C40B	90.9 (4)
C33A—Sb1A—C39A—C44A	56.4 (4)	C27B—Sb1B—C39B—C44B	40.5 (4)
C27A—Sb1A—C39A—C44A	−50.6 (4)	C33B—Sb1B—C39B—C44B	140.5 (3)
Ru3A—Sb1A—C39A—C44A	−174.5 (4)	Ru3B—Sb1B—C39B—C44B	−81.9 (4)
C44A—C39A—C40A—C41A	−1.7 (8)	C44B—C39B—C40B—C41B	0.4 (7)
Sb1A—C39A—C40A—C41A	177.4 (4)	Sb1B—C39B—C40B—C41B	−172.2 (4)
C39A—C40A—C41A—C42A	0.9 (9)	C39B—C40B—C41B—C42B	0.7 (9)
C40A—C41A—C42A—C43A	0.9 (9)	C40B—C41B—C42B—C43B	−1.5 (9)
C41A—C42A—C43A—C44A	−1.9 (9)	C41B—C42B—C43B—C44B	1.1 (9)
C42A—C43A—C44A—C39A	1.1 (8)	C42B—C43B—C44B—C39B	0.1 (8)
C40A—C39A—C44A—C43A	0.7 (8)	C40B—C39B—C44B—C43B	−0.9 (7)
Sb1A—C39A—C44A—C43A	−178.4 (4)	Sb1B—C39B—C44B—C43B	172.2 (4)

### Hydrogen-bond geometry (Å, °)

**Table:**

Cg1, Cg2 and Cg3 are the centroids of the C21B–C26B, C39B–C44B and C15A–C20A benzene rings, respectively.

**Table:**

*D*—H···*A*	*D*—H	H···*A*	*D*···*A*	*D*—H···*A*
C18A—H18A···O7B^i^	0.93	2.55	3.337 (7)	142
C34A—H34A···O2A	0.93	2.56	3.375 (7)	147
C34B—H34B···O8B^i^	0.93	2.57	3.414 (7)	151
C5B—H5BA···Cg1^ii^	0.93	3.00	3.723 (9)	136
C25B—H25B···Cg2^iii^	0.93	2.95	3.856 (8)	166
C43A—H43A···Cg3^iii^	0.93	2.91	3.624 (6)	135

Symmetry codes: (i) −*x*+1, −*y*+1, −*z*+1; (ii) −*x*, −*y*, −*z*+1; (iii) *x*−1, *y*, *z*.
